# Development of an effective two-equation turbulence modeling approach for simulating aerosol deposition across a range of turbulence levels

**DOI:** 10.1016/j.jaerosci.2023.106262

**Published:** 2024-01

**Authors:** Hasan Jubaer, Morgan Thomas, Dale Farkas, Arun V. Kolanjiyil, Mohammad A.M. Momin, Michael Hindle, Worth Longest

**Affiliations:** a Department of Mechanical and Nuclear Engineering, Virginia Commonwealth University, 401 West Main Street, P.O. Box 843015, Richmond, VA, 23284-3015, USA; b Department of Pharmaceutics, Virginia Commonwealth University, Richmond, VA, USA

**Keywords:** CFD, Aerosol, Turbulence, Particle deposition, Air-jet DPI, Preterm infant, Respiratory drug delivery

## Abstract

Pharmaceutical aerosol systems present a significant challenge to computational fluid dynamics (CFD) modeling based on the need to capture multiple levels of turbulence, frequent transition between laminar and turbulent flows, anisotropic turbulent particle dispersion, and near-wall particle transport phenomena often within geometrically complex systems over multiple time scales. Two-equation turbulence models, such as the k−ω family of approximations, offer a computationally efficient solution approach, but are known to require the use of near-wall *(NW) corrections* and eddy interaction model *(EIM) modifications* for accurate predictions of aerosol deposition. The objective of this study was to develop an efficient and effective two-equation turbulence modeling approach that enables accurate predictions of pharmaceutical aerosol deposition across a range of turbulence levels. Key systems considered were the traditional aerosol deposition benchmark cases of a 90-degree bend (Re=6,000) and a vertical straight section of pipe (Re=10,000), as well as a highly complex case of direct-to-infant (D2I) nose-to-lung pharmaceutical aerosol delivery from an air-jet dry powder inhaler (DPI) including a patient interface and infant nasal geometry through mid-trachea (500<Re<7,000). Of the k−ω family of models, the low Reynolds number (LRN) shear stress transport (SST) approach was determined to provide the best agreement with experimental aerosol deposition data in the D2I system, based on an improved simulation of turbulent jet flow that frequently occurs in DPIs. Considering NW corrections, a new correlation was developed to quantitatively predict best regional values of the $y^{+}\;limit$, within which anisotropic NW turbulence is approximated. Considering EIM modifications, a previously described drift correction approach was implemented in pharmaceutical aerosol simulations for the first time. Considering all model corrections and modifications applied to the D2I system, regional relative errors in deposition fractions between CFD predictions and new experimental data were improved from 19–207% (no modifications) to 2–15% (all modifications) with a notable decrease in computational time (up to ∼15%). In conclusion, the highly efficient two-equation k−ω models with physically realistic corrections and modifications provided a viable, efficient and accurate approach to simulate the transport and deposition of pharmaceutical aerosols in complex airway systems that include laminar, turbulent and transitional flows.

## Highlights

• Two-equation turbulence models require both *NW corrections* and *EIM modifications*.
• LRN k−ω SST model performs well with dry powder inhaler systems.
• NW corrections: New quantitative correlation for predicting best $y^{+}$ limits.
• EIM modifications: Drift flux correction is important in pharmaceutical aerosol systems.
• k−ω models are an accurate and efficient option for pharmaceutical aerosol simulations.

## Graphical abstract

**Figure:**
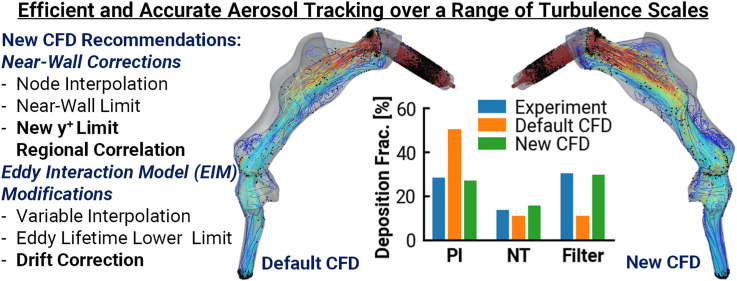

## 1 Introduction

In the field of pharmaceutical aerosol delivery, Computational Fluid Dynamics (CFD) provides an effective approach to capture the transport of particles and droplets within inhaler devices, patient interfaces (PIs), and the respiratory airways (Bass et al., 2021; Capecelatro et al., 2022; Chen et al., 2021; Longest et al., 2019; Longest & Holbrook, 2012; Ruzycki et al., 2013). An accurate, i.e., well-validated, CFD model can be applied to design and optimize efficient inhalation devices and PIs (Bass et al., 2021, 2022; Bass & Longest, 2020; Longest et al., 2015; Longest & Farkas, 2019). These models can also be used to develop effective aerosol delivery strategies for maximizing the lung delivery efficiency of inhaled pharmaceutical aerosols and for possibly targeting the region of deposition within the lungs (Longest et al., 2016; Longest & Holbrook, 2012).

When using CFD simulations to explore the transport dynamics of pharmaceutical aerosols from the aerosolization device to within the lungs, a common challenge is the construction of a model to capture the flow field turbulence and the associated impact of turbulence on particle or droplet motion and deposition (Longest et al., 2019; Longest & Holbrook, 2012; Ruzycki et al., 2013). With most aerosol generation devices, and especially with dry powder inhalers (DPIs), turbulence levels are very high within the device, which helps to form the aerosol, and within the PI (Longest & Farkas, 2019). This high turbulence is then passed into the extrathoracic airways and can be further magnified by the PI (Bass, Boc, et al., 2019) and the larynx (Lin et al., 2007; Xi et al., 2008). Within all of these regions, turbulence can significantly increase aerosol deposition through turbulent dispersion, which involves random turbulent fluctuations (or eddies) moving particles in all directions including towards wall boundaries. Turbulent dispersion can enhance the deposition of particles that are already moving near a wall boundary due to particle inertia, i.e., turbulence enhanced particle impaction. Alternatively, turbulent dispersion can create significant deposition in cases where particle-wall impaction would otherwise be absent, as with a long straight vertical section of conduit, i.e., pure turbulent dispersion. Even more challenging, the transition of turbulence physics from high to low turbulence levels and then to laminar flow is extremely difficult to predict (Durbin, 2018; Langtry & Menter, 2009; Menter et al., 2004), particularly for RANS models, because the closure coefficients are tuned to match flow characteristics from established fully turbulent or homogenously turbulent flows.

Turbulence models of the flow field are typically classified as direct numerical simulation (DNS), Large Eddy Simulation (LES), and Reynolds-averaged Navier-Stokes (RANS) approaches, which are reviewed elsewhere (Argyropoulos & Markatos, 2015; Lin et al., 2007; Luo et al., 2004; Zhang & Kleinstreuer, 2003, 2011b). Briefly, the DNS approach fully resolves all turbulence scales at a high computational expense, which has led to only a limited number of studies (e.g. Lin et al., 2007; Nicolaou & Zaki, 2013; Stylianou et al., 2016) that implement this approach. By contrast, LES resolves only the large-scale eddies and uses sub-grid scale models to approximate small eddy effects. LES proves more computationally affordable than DNS and, as a result, multiple studies have employed LES to investigate particle deposition patterns and flow field characteristics in the airways (e.g. Abdelsamie et al., 2023; Cui & Gutheil, 2011; Dehbi, 2011; Ilie et al., 2008; Rajaraman et al., 2023; Talaat et al., 2022). In contrast, the Reynolds-averaged Navier-Stokes (RANS) approach approximates turbulent effects with time-averaged transport equations. For instance, the *two-equation* models under this category use equations for turbulent kinetic energy (k) and a dissipation term $\left(\omega \;\text{or}\;\varepsilon \right)$ to estimate turbulent features. The RANS approach is the least-detailed of the three approaches, but is also the most computationally efficient which has led to its widespread use throughout the field of aerosol transport modeling.

Studies that have compared turbulence modeling approaches have generally found that the more complex models demonstrate superior accuracy in capturing local flow features, especially when considering time dependence. However, the considerable increase in computational time and power to attain this accuracy is well recognized. Zhang and Kleinstreuer (2011b) reported a 100-fold increase of computational resources required by LES over RANS models for a simulation of human upper airways. It should be noted that comparisons of computational requirements reported in the literature are strongly dependent on the specific flow type and the fraction of the range of turbulence scales resolved by the employed LES model. Recently, Abdelsamie et al. (2023) noted in a detailed comparative study of laryngeal flow that a RANS model required seven times less computational power than an LES simulation, with LES requiring 170 times less power than a DNS simulation. They also highlighted that RANS predicted the overall flow features averaged in time and space reasonably well, despite ultimately proving less accurate in predictions of turbulent quantities and fluctuations. In this context, it may be noteworthy that the disparities in the detailed flow features predicted by two-equation RANS models vs. LES or DNS models do not always decrease the accuracy of particle transport and deposition predictions. In fact, despite their less-accurate solutions for some flow features, two-equation models can be successful in predicting aerosol transport in the airways (Longest et al., 2019; Longest & Holbrook, 2012; Zhang & Kleinstreuer, 2011a). Nevertheless, *in silico* studies typically strive to achieve as accurate a flow field solution as possible. To this end, it is also important to note that recently there has been a significant amount of research on hybrid RANS-LES approaches that use a RANS model in the near-wall (NW) region and LES in the outer region (Davidson & Peng, 2003; Fröhlich & von Terzi, 2008). This reduces the need for fine mesh resolution in the NW region, saving computational resources (Argyropoulos & Markatos, 2015). Moreover, the flow field predictions by the approaches that hybridize RANS and LES models were often reported to be more accurate than those by RANS models (Heinz, 2020). While this approach has not yet been used in respiratory flow simulations, it certainly has the potential to be useful for optimizing computational efforts and accurately resolving turbulent flow fields.

In selecting a turbulence model for predicting the deposition of pharmaceutical aerosols in a manner that balances efficiency and accuracy, our group has frequently taken the position that:

(i) The solution efficiency of RANS models should not be overlooked, especially when considering low Reynolds number (LRN) turbulence and transitional flows, multiple time scales ranging from vortex shedding time periods (∼0.01 s) through full inhalation cycles (∼5 s), and the complexity of geometries that may include the inhaler, extrathoracic airways and lungs (Dutta et al., 2020; Longest, Tian, Walenga, & Hindle, 2012; Xi & Longest, 2009).
(ii) RANS models may provide adequately resolved flow and turbulence fields in steady state and transient simulations that enable high quality matches with experimental aerosol deposition data (Bass et al., 2019, 2022; Longest et al., 2015, 2016; Longest & Hindle, 2009b; Tian et al., 2011b, 2015; Walenga & Longest, 2016; Xi & Longest, 2009).
(iii) RANS flow field models require corrections for accurately predicting turbulent particle deposition with an emphasis on NW conditions, including anisotropic turbulence (Bass & Longest, 2018; Longest & Xi, 2007; Walenga & Longest, 2016).
(iv) Selection of a meshing scheme and adequate resolution of the NW mesh is highly important for the successful use of all turbulence models, including the RANS approach (Bass & Longest, 2018; Thomas & Longest, 2022).

Following these principles, our group has successfully employed the LRN k−ω model to accurately predict the deposition of particles in the upper respiratory airways, which has been validated with data from both *in vitro* (Longest, Tian, Walenga, & Hindle, 2012; Longest & Hindle, 2009a; Longest & Vinchurkar, 2007; Longest & Xi, 2007) and *in vivo* (Longest et al., 2016; Tian et al., 2015) experiments. These simulations have considered pharmaceutical aerosols from multiple inhaler types including DPIs (Longest et al., 2012a, 2012b; Tian, Longest, Su, Walenga, & Hindle, 2011), metered dose inhalers (MDI) (Longest, Tian, Walenga, & Hindle, 2012; Walenga et al., 2013; Walenga & Longest, 2016), and soft mist inhalers (Delvadia et al., 2013; Longest et al., 2009; Longest & Hindle, 2009a). For instance, Longest, Tian, Walenga, and Hindle (2012) demonstrated that the mouth-throat deposition predicted by the LRN k−ω model with NW corrections was in agreement with *in vitro* experimental data for realistic pharmaceutical aerosols, considering the effect of polydisperse aerosol size, transient inhalation over an approximately 5 s period, and turbulence for MDI and DPI devices, with relative differences of less than 10%.

In simulating pharmaceutical aerosols, there is no universal recommendation for choosing a specific RANS variant, so the choice is usually made on a case-specific basis. Nevertheless, the most commonly used two-equation model variant may be the k−ω turbulence model, which was originally developed by Wilcox (Wilcox, 1988, 2006). Since laminar, transitional, and turbulent flows are expected to occur in human airways, the standard the k−ω turbulence model augmented by the low Reynolds Number (LRN) correction (Wilcox, 2006) has proven useful for modeling regions that include the onset and dissipation of turbulence at low magnitude (Longest et al., 2019; Longest & Holbrook, 2012; Zhang & Kleinstreuer, 2003). Advantages of the LRN k−ω approach include an accurate prediction of the flow variables such as pressure drop, velocity profiles, and shear stress by efficiently implementing a damping coefficient for the eddy viscosity, which allows it to be used in simulations ranging across laminar, transitional and turbulent flow regimes (Ghalichi et al., 1998; Wilcox, 2006). Consequently, the LRN k−ω turbulence model has proven effective in predicting particle deposition in the upper airways with satisfactory accuracy in many studies (e.g. Ahookhosh et al., 2021; Bass, Boc, et al., 2019; Inthavong et al., 2010; Longest & Hindle, 2009b; Pourmehran et al., 2016; Tian, Longest, Su, Walenga, & Hindle, 2011; Tian et al., 2015; Varghese & Frankel, 2003; Xi & Longest, 2009; Zhang et al., 2005; Zhang & Kleinstreuer, 2003; Zhang & Kleinstreuer, 2004). Another variant of two-equation RANS models is the k−ε turbulence model, which is widely used in various engineering applications involving fully turbulent flows due to its strength in representing flow fields of high eddy viscosity. Still, Zhang and Kleinstreuer (2003) found that the k−ε model failed to perform well in laminar and transitional flow regimes, making it unsuitable for the modeling of flow dynamics across the human airways.

Taking into account the distinct advantages and disadvantages of two-equation models, Menter (1994) adopted a practical approach that combined the k−ε and k−ω models using a blending function. Further improvement was made to the original baseline formulation of the proposed model by incorporating the transport of principal turbulent shear stress. The resultant model is known as the k−ω Shear Stress Transport (SST) model, which has proven suitable for simulation of airflow in human upper airways in some applications (e.g. Elcner et al., 2016; Gurumurthy & Kleinstreuer, 2021; Jayaraju et al., 2007; Kolanjiyil & Kleinstreuer, 2016, 2017). A recent study by Chen et al. (2023) assessed the ability of various turbulence models to predict the experimentally measured pressure and velocity of airflow in pediatric airways. Among the RANS models, the LRN k−ω SST model exhibited the greatest potential for use in pediatric respiratory studies, as it displayed the closest results to those obtained through LES simulations at a fraction of the computational cost.

The challenge of turbulence model selection is compounded by the fact that the chosen approach will impact the way that particles are influenced by turbulent dispersion. Regardless of the selected variant, when two-equation models are employed the Eulerian-Lagrangian approach is typically used to predict aerosol transport. The approach consists of solving the flow field (Eulerian view) and subsequently injecting particles that are individually tracked until they evaporate, escape from the domain, or deposit on a boundary. Discrete phase models (DPM) simulate the particle motion and include forces acting on the particle, as well as a particle dispersion model, also known as the eddy interaction model (EIM), which accounts for the effect of turbulent fluctuations on the particle trajectory. As the name implies, the EIM approximates the interaction of individual particles with successive discrete eddies that have length, velocity, and characteristic lifetimes and are derived from the Eulerian flow field (Matida et al., 2004). The EIM stochastically recreates eddy characteristics using the local mean fluid velocity and turbulence quantities such as turbulent kinetic energy (TKE, k) and specific dissipation rate (SDR, ω) to mimic the impact of an eddy on particle transport. This process results in a discrete random walk (DRW) of the particle path (MacInnes & Bracco, 1992).

In using the Eulerian-Lagrangian approach, a major limitation is that all two-equation models implement the assumption of isotropic turbulence. Matida et al. (2000) established that the isotropic turbulence assumption, when applied in a NW region, causes an overprediction of particle deposition. Hence, Matida et al. (2004) proposed a NW anisotropic correction that has improved accuracy. However, the range within which the turbulence correction was applied $\left(1<\;y^{+}\;limit\;<20\right)$ varied for different flow rates and turbulent conditions. A method does not currently exist to quantitatively predict the appropriate $y^{+}\;limit$. Many researchers (e.g. Chen et al., 2017; Huang et al., 2022; Rajendran et al., 2021; Xi et al., 2011; Yan et al., 2019; Yousefi et al., 2017) including our group have implemented this correction but the limit must always be determined based on the specific case, and comparison to experimental data, i.e., tuning the model. Therefore, in a case which contains abruptly changing flow parameters or irregular geometries, a strategy to resolve the problem is unavailable at present. Furthermore, there is no work that considers similar turbulence dampening requirements in transitional flows. Such transitional turbulent flows dominate in respiratory drug delivery because all flowrates are subject to the tidal breath cycle.

One further limitation is that the DRW model was developed for homogeneous turbulent flows. In such regions, there is little or no TKE gradient and the model is consequently not designed to compensate for these gradients when they occur. Unfortunately, this leads to over-concentration of particles with low Stokes (Stk) numbers in zones of high TKE gradient and consequently causes an over-prediction of deposition in many cases (Mofakham & Ahmadi, 2020). Mofakham and Ahmadi (2020) have proposed strategies for improving this limitation but these have not been utilized in respiratory drug delivery simulations. In the same study, the authors showed that the Continuous Random Walk (CRW) model also has qualities which overcome the listed challenges. In the current study, we focus on corrections of the DRW model, which is the basis for the popular Ansys Fluent CFD package.

Our group has focused on the use of the k−ω (standard; LRN; SST) models in the simulation of pharmaceutical aerosols in devices, PIs, and respiratory airways. A major focus of this work has been the development of NW corrections to the flow field and particle transport variables in order to address some of the issues identified above. These NW corrections have typically included approximations for NW turbulence, interpolation of NW properties within control volumes from nodal values, and damping wall-normal velocity as particles approach wall boundaries. In earlier studies with relatively high turbulence, these NW corrections proved useful in validating CFD predictions of aerosol depositions with experimental data (Dutta et al., 2020; Longest et al., 2007, 2012b, 2013, 2015; Longest & Farkas, 2019; Longest & Hindle, 2009b; Tian et al., 2011a, 2015; Walenga & Longest, 2016; Xi & Longest, 2007, 2009). We then refined these techniques for lower level turbulence and transitional flow, first in a benchmark curved tube geometry (Bass & Longest, 2018) and then in an infant airway model (Bass, Boc, et al., 2019). These modifications were useful in the development of pediatric DPIs (Bass & Longest, 2020) and in understanding the impact of nasal prongs on infant respiratory drug delivery (Bass et al., 2022). Nevertheless, it was observed that with transitional turbulence, the selected value of the $y^{+}\;limit$ was very important. In addition, these previous systems may not have adequately addressed turbulent particle deposition by pure turbulent dispersion. In systems with a wide range of turbulence levels combined with the potential for pure turbulent dispersion and impaction, an expansion of previous NW corrections together with new EIM modifications is needed.

The objective of this study is to develop an efficient and effective two-equation turbulence modeling approach that enables accurate predictions of pharmaceutical aerosol deposition across a range of turbulence levels (including fully turbulent, transition from turbulent-to-laminar and laminar flow) for application to a new air-jet-based DPI system. Mechanisms responsible for aerosol deposition in this system include both impaction, enhanced by turbulent dispersion, and pure turbulent dispersion. To maintain high CFD solution efficiency, the turbulence model is based on the RANS two-equation approach. The accuracy of these models for predicting aerosol deposition will be improved through the use of multiple *NW corrections* and *EIM modifications*. As previously described and updated in this study, NW corrections include the use of anisotropic turbulence, interpolation of intra-control-volume variables, and a NW limit. In this study the EIM modifications include use of interpolated fluctuation velocity and TKE, a lower limit on eddy lifetime, and a drift correction term (Bocksell & Loth, 2001, 2006; Mofakham & Ahmadi, 2020). The pharmaceutical aerosol system selected is an air-jet DPI (Farkas et al., 2018a; Farkas et al., 2018b) for administering small-particle aerosols to infants (Bass, Boc, et al., 2019; Howe et al., 2022a, 2022b) via the nose-to-lung (N2L) route. To ensure that deposition mechanisms are accurately captured across a range of turbulence levels, additional benchmark systems are also considered, including a curved tube (deposition due to impaction enhanced by turbulent dispersion) and a vertical straight pipe (dispersion due purely to turbulent dispersion). The overall goal of the final model is to capture experimentally measured aerosol deposition within different regions of the infant air-jet delivery system in a manner that falls within the standard deviation bounds of concurrent experimentally measured data. Ideally, the absolute and relative errors of comparisons between CFD predictions and experimental data within each region will be less than 5% and 15%, respectively.

## 2 Methods

### 2.1 Study overview

As described, this study seeks to develop an effective RANS two-equation modeling approach that enables accurate predictions of aerosol deposition across a range of Reynolds numbers (Re) and test geometries. The test systems selected include the benchmark cases of turbulent particle deposition in a curved tube (Pui et al., 1987), a vertical straight pipe (Liu & Agarwal, 1974), and pharmaceutical aerosol delivery to an infant using a new air-jet DPI and the N2L approach. The curved tube geometry (Re=6,000) was introduced to capture turbulence assisted impaction deposition and the vertical straight pipe (Re=10,000) was intended to capture deposition due to purely turbulent dispersion. The infant air-jet system includes both of these previously mentioned turbulent deposition mechanisms as well as high initial turbulence in the PI followed by diminishing turbulence and transitional flow in the infant's nasal region (approximately 500<Re<7,000). Two forms of the PI were considered, which are a gradual and a rapid expansion from the high velocity air jet to the single prong interface. These two configurations include elements of gradual flow separations and free shear flow, which are both challenging to accurately capture with turbulence models.

The LRN k−ω and LRN k−ω SST turbulence models were considered in addition to both existing and new NW corrections and two EIM modifications, all of which are described in subsequent sections. This work builds on the existing turbulence model enhancements of Bass et al. (Bass, Boc, et al., 2019; Bass & Longest, 2018) (referred to as the Bass model) and Thomas and Longest (2022) (referred to as the Thomas model). For the benchmark test cases of the curved tube and vertical pipe, CFD results are compared with existing experimental data from the literature, as outlined below. For the case of pharmaceutical aerosol delivery to infants, new experimental data was generated and is also reported in this study.

### 2.2 Selected test systems

#### 2.2.1 Benchmark systems

**Fig. 1:**
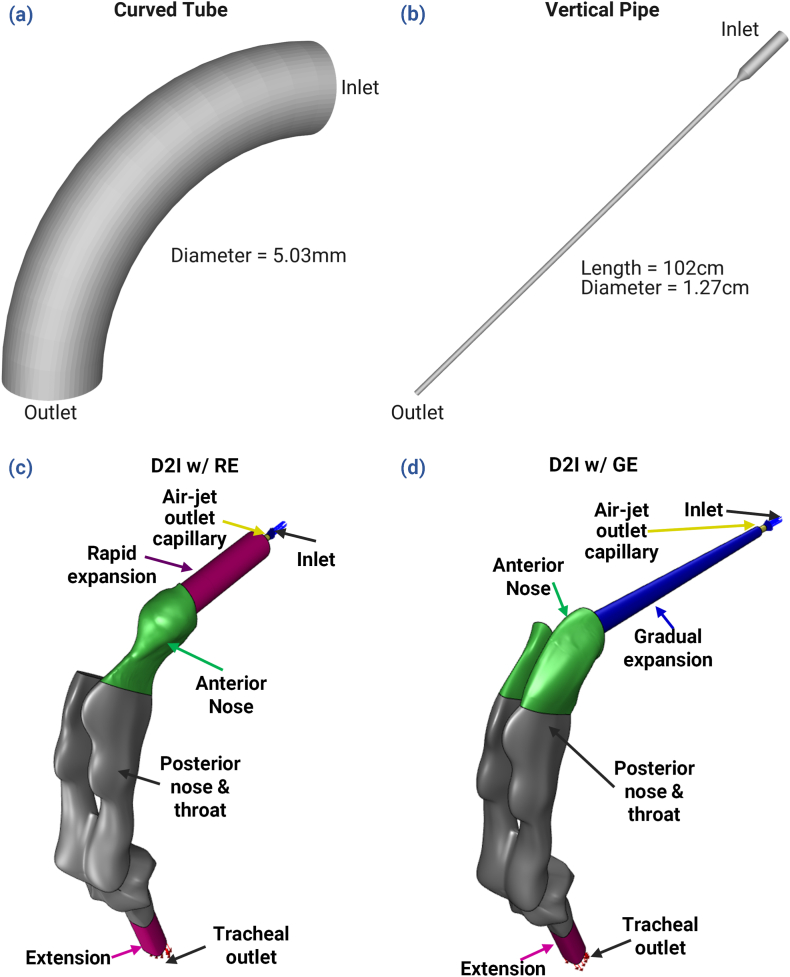
Calculation domains for the benchmark geometries: (a) curved tube, and (b) vertical pipe. D2I calculation domains (interior fluid space) for N2L delivery systems with different patient interfaces: (c) a rapid expansion (D2I w/RE), and (d) gradual expansion (D2I w/GE).

Pui et al. (1987) analyzed and created detailed benchmark data of aerosol deposition for the 90°-bend geometry using configurations of variable cross section and flowrate. The current study uses the configuration which consists of a 5.03 mm tube diameter, a 5.7 bend ratio (radius of bend over radius of cross section), and a Re of 6,000 (see Fig. 1a) (0.03≤Stk≤1.46).

Liu and Agarwal (1974) executed a benchmark aerosol deposition experiment based on a vertical glass pipe with 1.27 cm diameter and a length of 102 cm (see Fig. 1b). This study uses the case where Re was 10,000 for comparison (0.3≤Stk≤32).

#### 2.2.2 Direct-to-infant (D2I) aerosol delivery systems

The D2I aerosol delivery systems (see Fig. S1 in the supplemental materials) consist of an air-jet DPI device, a patient interface, and a Nose-Throat (NT) model of a preterm infant (see Fig. 1c and d). The outlet capillary from the air-jet DPI device (Farkas et al., 2018a; Farkas et al., 2018b) has a diameter of 0.89 mm. A gradual expansion (GE) or rapid expansion (RE) chamber was attached to the outlet capillary. A curved nasal prong was connected to the outlet of the GE and RE to direct the aerosol into the infant's nose. Further details on aerosol delivery to infants using the D2I approach are provided elsewhere (Howe et al., 2022a, 2022b).

The NT model used in this study was developed by our group and reported in Bass et al. (2022) from CT scans of a 28-week old preterm infant that were provided by Seattle Children's Hospital (Youngquist et al., 2013). The original CT scans did not include the laryngeal region, which is known to be an important component of flow development entering the upper airways (Xi et al., 2008). Therefore, the laryngeal region and a part of the trachea were extracted from a 6-month-old NT model (Bass, Boc, et al., 2019), scaled to dimensions consistent with a 1,500 g preterm infant, and smoothly coupled to the preterm NT model to form a full model from nares through the middle passage, nasopharynx, larynx, and a section of the trachea. Further details on the NT model development can be found elsewhere (Bass et al., 2022).

The NT model was connected to an infant test lung (Michigan Instruments Inc. Model 1601, Grand Rapids, Michigan). The compliance was set to 1 mL/cm H_2_O and a total resistance of 80 cm H_2_O/L/s was calculated after adding a resistor between the filter and infant lung simulator. A total inhalation volume of 10 mL was verified using flow profiles obtained from a Sensirion SFM3400 flow meter (Sensirion AG, Stafa, Switzerland).

### 2.3 CFD simulations

#### 2.3.1 Computational domains and boundary conditions

In order to represent the experiment by Pui et al. (1987) and compare with the detailed benchmark data of aerosol deposition for the curved tube (see Fig. 1a), a constant velocity condition of 17.42 m/s was applied at the inlet of a numerical extension (length of 10 times the inlet diameter) to the curved tube and a pressure outlet boundary condition was applied at the outlet of an additional extension of the same length (see Thomas & Longest, 2022 for details). Similarly, in order to mimic the experiment of the vertical pipe benchmark case, flow was solved in a straight pipe using a mass-flow inlet of 86.36 LPM (L/min). The straight pipe outlet used the pressure-outlet boundary condition and the values for velocity components, TKE, and SDR were recorded as profile data for use as inlet conditions for the geometry shown in Fig. 1b, which utilized a pressure outlet. All walls in the two benchmark domains were set to trap particles on contact.

The computational domains for D2I nasal aerosol delivery setups consisted of two main components that were assembled together: the PI and the preterm NT model (see Fig. 1c & d). The PI comprises the nasal prong and the flow pathway that connects the air-jet DPI device to the nasal prong. There are two types of PI: Gradual expansion (GE) and Rapid expansion (RE). As the air-jet DPI device was not a component of the CFD-model, the jet inlet to the PI was replicated using part of its outlet capillary (Air-jet outlet capillary in Fig. 1c & d) and was included in the model. This outlet capillary has a diameter 0.89 mm, and its length is three times the diameter. The NT model contains the anterior as well as posterior nose, throat, and the tracheal outlet to the deposition filter. An additional numerical extrusion (with a length three times the diameter) was added to the tracheal filter connection outlet.

Meshes for all computational domains were generated using Ansys Fluent Meshing v2022R2 (Ansys Inc., Canonsburg, PA, USA), following the best practices established by previous publications (Bass & Longest, 2018; Thomas & Longest, 2022). For further information on meshing strategies, resolution, and convergence readers are referred to the supplemental information.

The D2I CFD models were configured to mimic the flow in and out of the experimental model, which is comprised of a single inlet and a single outlet. A velocity inlet boundary condition was applied at the inlet of the air-jet outlet capillary and an outflow boundary condition was applied at the filter connection outlet. The inlet conditions such as the profiles of the individual velocity components, and turbulence parameters (k & ω), at the inlet face to the outlet capillary, were set by means of a fully developed flow profile derived from a separate CFD simulation. To obtain this inlet profile, the flow field for a mass flow rate of 3.9 LPM (Q90 of experimentally measured device flow rate, where Q90 refers to the volumetric flowrate below which 90% of the rapidly sampled data of the measured flowrate lie) through a long inlet extension (length 30 times the diameter) with identical mesh resolution was separately solved.

Particles were set to be trapped at the wall boundaries of the D2I flow domain. The only exception was the wall of the metal outlet capillary which was set to reflect the particles on impaction. This setting was selected based on the experimental observation of no particle deposition on the capillary tube wall due to a high shear stress on the smooth metal wall surface. Following the recommendation of Bass et al. (2022), a wall roughness value of 50 μm was applied at the NT wall surfaces to account for the effect of surface characteristics on the flow field in the NW region and its impact on particle transport and deposition.

#### 2.3.2 Numerical methods and solver settings

The commercial package Ansys Fluent v2022R2 (Ansys Inc., Canonsburg, PA, USA) was employed to solve all governing mass and momentum conservation equations. The theoretical summary of the relevant governing transport equations is available in our previously published works (e.g. Longest et al., 2006; Longest et al., 2007; Longest & Xi, 2007). Multiple user defined functions (UDFs) were utilized to modify the default models provided by the commercial package for discrete phase transport calculations.

The model and solver settings were applied according to the best practices established for airway simulations with unstructured mesh topology (Bass, Boc, et al., 2019; Bass & Longest, 2018). The pressure-based 3D double precision solver was utilized to solve the flow equations. All transport equations were spatially discretized ensuring second order accuracy, while the gradient discretization was achieved via the Green-Gauss Node-based method. Pressure-velocity coupling was captured with the SIMPLEC algorithm. Convergence of all steady state flow field solutions was assumed when each residual had decreased by at least five orders of magnitude, and no further changes were detected in the velocity magnitude and TKE volume averages, which were computed across the entire domain.

Particle trajectory was integrated over the Lagrangian force balance using Ansys Fluent's DPM:

(1) $$\frac{du_{p}}{dt}=\frac{f}{\tau_{p}}\left(u_{f}-u_{p}\right)+\frac{g\;\left(\rho_{p}-\rho_{f}\right)}{\rho_{p}}$$

where $u_{p},d_{p}$ and $\rho_{p}$ are particle (discrete phase) velocity, diameter, and density; while $u_{f}$ and $\rho_{f}$ represent fluid (continuous phase) velocity and density. The particle relaxation time was expressed as $\tau_{p}=\frac{\rho_{p}d_{p}^{2}}{18\mu_{f}}$. It is noted that the drag factor f, representing the ratio between the drag coefficient and Stokes drag, was calculated as (Morsi & Alexander, 1972):

(2) $$f=\frac{C_{D}Re_{p}}{24}=\frac{Re_{p}}{24}\left(a_{1}+\frac{a_{2}}{Re_{p}}+\frac{a_{3}}{Re_{p}^{2}}\right)$$

The coefficients $a_{i}$ are constants proposed for smooth spherical particles over the range of particle Reynolds number suitable in the current study, $0\leq Re_{p}\leq 100$.

Additional potentially relevant forces such as Brownian motion (for the submicrometer particles), lift, and particle rotation were screened and found to have a negligible contribution toward correcting the initial errors observed between the experiments and baseline CFD predictions of aerosol deposition. While electrostatics may also play a role, sufficient data was not available on the complex nature of the aerosol charge that would be required to assess this effect.

To integrate the equation of particle motion, automated tracking was activated, which automatically switches between a lower-order implicit scheme for particles close to hydrodynamic equilibrium and a higher-order Runge-Kutta scheme for particles far from hydrodynamic equilibrium. This improves calculation efficiency while maintaining accuracy (Ansys Fluent, 2022). In addition, the tolerance of the solution was specified by setting an accuracy control of $10^{-6}$ with a maximum of 20 refinements of the time step size per particle integration step.

To account for the turbulent dispersion of particles, instantaneous fluid velocity $\left(u_{f}\right)$ was expressed as a combination of the fluctuating component of the fluid phase velocity $\left(u_{f}^{\prime }\right)$ and the time-averaged velocity $\left({\bar{u}}_{f}\right)$ as in Eq. (1):

(3) $$u_{f}={\bar{u}}_{f}+u_{f}^{\prime }$$

These velocity components were then utilized in a standard discrete random walk (DRW) model first introduced by Gosman and loannides (1983). This EIM is currently common practice to capture turbulent particle dispersion in Eulerian-Lagrangian simulations and is available as a default option in Ansys Fluent. However, due to extensive modifications employed in this study, this standard EIM was implemented by utilizing UDFs (with further details provided in subsequent sections). It is also noted that some simulation cases included DRW effects (i.e., particle dispersion) and some did not (i.e., no dispersion).

#### 2.3.3 Turbulence models

This study compares the LRN k−ω and the LRN k−ω SST turbulence models in order to identify best practices when modeling laminar, transitional, and turbulent flow fields in the systems of interest. In the LRN k−ω case, shear flow corrections (SFC) were included by default in order to enhance the dissipation of TKE and damp the dissipation of SDR according to the original k−ω approach (Wilcox, 2006). The exception is the curved tube case, where Thomas and Longest (2022) found that SFC were unnecessary due to the absence of free shear flow. When SFC corrections are used, empirical equations established by Wilcox are applied to determine the coefficients used in the calculations of the dissipation of TKE and SDR, which are otherwise set to 1. The Low Reynolds Number (LRN) corrections ensure that the model achieves a) asymptotic consistency for TKE and SDR as y→0, and b) decoupling of the TKE and SDR equations in laminar and transitional flows, defined by appropriate Reynolds Numbers for a flat plate (Wilcox, 2006). Considering the k−ω SST model, Menter (1994) proposed the “Baseline model” by merging both k−ε and k−ω models with a blending function and then later incorporated the transport of principal shear stress in adverse pressure gradient boundary-layers to formulate the final k−ω SST model. Hence, the designation LRN k−ω SST refers to the SST variant of the two-equation k−ω model including the LRN correction. Note that the employed k−ω SST model should not be confused with the four-equation transition SST model developed by Langtry and Menter (2009).

#### 2.3.4 Near-wall corrections

Two-equation turbulence models such as k−ω include the limiting assumption that turbulence is isotropic, i.e., having the same magnitude in all directions. The fluctuating component shown in Eq. (3) is then estimated to be:

(4) $$u_{f}^{\prime }=v_{f}^{\prime }=w_{f}^{\prime }=G_{i}\;\sqrt{\frac{2}{3}k}$$

Here, $G_{i}$ is a random number with Gaussian distribution, a mean of 0, and a standard deviation of 1 that represents the varying intensity of turbulent eddies. This assumption is computationally efficient but it complicates particle transport predictions in domains where anisotropy is expected. One such domain is the turbulent boundary layer, where eddies stretch in the presence of NW shearing effects. Matida et al. (2004) used DNS results in a simple channel flow to establish a correlation between the velocity of wall-directed turbulent fluctuations ($u_{n}^{\prime }$) and TKE (k) based on the dimensionless particle height from the wall ($y^{+})$ and a random number with Gaussian distribution ($G_{n}$):

(5) $$u_{n}^{\prime }=G_{n}\left(1-e^{-0.02y^{+}}\right)\sqrt{\frac{2}{3}k}$$

This correlation was developed using the functions introduced by Wang and James (1999). The bracketed term exponentially damps wall-directed fluctuation velocity as a particle approaches a boundary wall. Matida et al. (2004) showed that the correlation applied in channel flow up to a particle height of $y^{+}≅80$, but this upper limit is not confirmed for non-channel flows. Therefore, the present study applies the correlation at particle heights up to the $y^{+}$ limit, which is varied between the cases..

Very close to wall boundaries, the physics of particle-wall interactions become highly complex. First, hydrodynamic interactions occur between the particle and wall, which are not typically included in standard discrete element tracking models (Longest et al., 2004). Secondly, the presence of the particle significantly disturbs the local flow, especially the wall-normal fluid velocity component, making it difficult to predict. As a computationally efficient approximation of these aspects, our group has previously introduced a tuning parameter called the NW limit, under which the wall-directed component of the continuous phase velocity is considered to be zero for the purpose of calculating particle trajectory (Bass & Longest, 2018; Walenga & Longest, 2016). Best values for NW limit currently prove to be case dependent, but do appear to correlate with particle size and Stokes number with a maximum on the scale of a few micrometers.

Interpolation of intra-control-volume variables is necessary, since by default, Ansys Fluent calculates particle trajectories using values of TKE and $u_{f}$ obtained from the center of the particle's host cell, neglecting intra-cell gradients. In order to include intra cell changes, cell-center values from the host and adjacent cells were interpolated to the particle's position using an inverse-distance-weighted average at every timestep. Previous publications from our group (Bass & Longest, 2018; Longest & Xi, 2007; Walenga & Longest, 2016), discussed justification of this interpolation method, which was formerly applied NW boundaries. NW corrections considered in this study, including intra-control volume interpolation (nodal interpolation), anisotropic turbulence ($y^{+}\;limit)$, and NW limit for different cases considered are summarized in Table 1.

**Table 1:** Summary of model features.

Case Name & number	*k-ω* Model		NW Corrections				Eddy Interaction Model		
	Base Model	SFC	Node interpolation		y^+^ limit [−]	NW limit [μm]	Variable inter-polation	Eddy lifetime lower limit	Drift corr-ection
			Velocity (up to)	*k* & *ω* (up to)					
1. No Dispersion LRN *k-ω*	LRN *k-ω*	Yes	N/A	N/A	N/A	N/A	No	Default	No
2. No Dispersion LRN *k-ω* SST	LRN *k-ω* SST	N/A	N/A	N/A	N/A	N/A	No	Default	No
3. Default LRN *k-ω*	LRN *k-ω*	Yes	N/A	N/A	0	0	No	Default	No
4. Default LRN *k-ω* SST	LRN *k-ω* SST	N/A	N/A	N/A	0	0	No	Default	No
5. Bass model	LRN *k-ω*	Yes	NW limit	y^+^ limit	60	2	No	Default	No
6. Thomas model	LRN *k-ω*	No	NW limit	y^+^ limit	60	0	No	Default	No
7. EIM Modifications w/o DC	LRN *k-ω* SST	N/A	No	No	0	0	Yes	Modified	No
8. EIM Modifications	LRN *k-ω* SST	N/A	No	No	0	0	Yes	Modified	Yes
9. Recommended	LRN *k-ω* SST	N/A	All domain	All domain	Correlated	0	Yes	Modified	Yes
10. Recommended nwlx	LRN *k-ω* SST	N/A	All domain	All domain	Correlated	x	Yes	Modified	Yes

#### 2.3.5 Eddy interaction model

One of the most common methods to model the effect of turbulent dispersion through an EIM on Lagrangian particle trajectories in two-equation flow fields is the DRW, which models turbulent dispersion as a set of discrete steps, each of which represents a single eddy and all of which represent a “walk” through the domain that mimics real turbulent dispersion. A single eddy consists of a fluctuation velocity $\left(u^{\prime }=G\sqrt{\frac{2}{3}k}\right)$ that varies with k as the particle travels through the flow field and where G is a vector of three components $G_{i}$ (defined above), which remain constant for the duration of the eddy's life ($t_{e}$). The eddy lifetime is set to twice the Lagrangian timescale ($t_{e}=2T_{l}$), which in the k−ω model is a function of ω (Ansys Fluent, 2022):

(6) $$T_{l}=\frac{c_{l}}{0.09\omega }$$

By default, Ansys Fluent simulations assume that the Lagrangian time scale constant ($c_{l}$) is 0.15, though others have used slightly different constants such as 0.2 (Kallio & Reeks, 1989). Additionally, eddy duration is limited by the eddy crossing time ($t_{cross}$), which accounts for a particle's ability to cross from a host eddy to a neighboring one. The crossing time compares the particle's relative velocity to the eddy's estimated length (${L_{e}=\left|u^{\prime }\right|t}_{e}$), and may be calculated as:

(7) $$t_{cross}=-\tau_{p}\left[1-\left(\frac{L_{e}}{\tau_{p}\left|u_{f}-u_{p}\right|}\right)\right]$$

where $\tau_{p}$ represents particle relaxation time. The remaining time spent in a single eddy is set to the minimum of the remaining eddy lifetime and the crossing time.

Three updates to the EIM – *variable interpolation, eddy lifetime lower limit,* and a *drift correction* – are considered in this paper for the purpose of maintaining high-accuracy implementation of the DRW. Section 2.3.4 describes the nodal interpolation of variables to particle position within cells, but formerly this interpolation has only been employed beneath the NW Limit. The first modification, i.e., *variable interpolation* is the removal of that cap, which allows intra-cell gradients to be considered throughout the domain. The second modification is an *eddy lifetime lower limit*. Ansys Fluent restricts particle timesteps by the remaining eddy lifetime, which asymptotes to zero near walls and results in timesteps as low as $1\times 10^{-9}\;s$ or less. This is unnecessarily small, so the modification places a lower limit on the dimensionless Lagrangian timescale $\left(T_{l}^{+}\right)$, where:

(8) $$T_{l}^{+}=T_{l}\frac{u_{*}}{\mu }$$

In this expression, $u_{*}$ is the friction velocity and μ is the dynamic viscosity. Kallio and Reeks (1989) placed this lower bound at ${\min(T}_{l}^{+})=10$, and to be conservative, the present study places that lower bound at ${\min(T}_{l}^{+})=3$.

The third EIM modification is a *drift correction* (DC), which addresses dispersive directionality. The direction of turbulent dispersion is set with the vector G and is reset once per eddy. Regions of high ω require shorter eddy lifetimes and cause a higher reset frequency, causing small particles, which are highly influenced by turbulent dispersion, to unrealistically “drift” towards such regions. To address this, Mofakham and Ahmadi (2020) proposed a DC term $\left(\bar{u_{i}^{\prime n+1}}\right)$ based on the work by Bocksell and Loth (Bocksell and Loth, 2001, Bocksell and Loth, 2006):

(9) $$u_{i}^{\prime n+1}=G_{i}\sigma_{i}+\bar{u_{i}^{\prime n+1}}$$

(10) $$\bar{u_{i}^{\prime n+1}}=\bar{u_{i}^{\prime n}}+\frac{1}{1+Stk}\sigma_{i}\frac{d\sigma_{i}}{dx_{j}}\Delta t$$

where $\sigma_{i}=\sqrt{\frac{2k}{3}}$, $\bar{u_{i}^{\prime n}}$ is reset to zero whenever $G_{i}$ is reset, Stk is the Stokes number defined as $\tau_{p}/T_{l}$, $\tau_{p}$ is the particle relaxation time, and Δt is the particle timestep. For the present study, the DC will be evaluated separately from the interpolation extent and eddy lifetime lower limit.

#### 2.3.6 Particle injection and deposition metrics

Particle injection details for the curved tube benchmark case can be found in Thomas and Longest (2022), but the number of particles injected per bin was 5,000, which aligns with the settings used in Bass and Longest (2018). In the vertical pipe, particles of diameter 1.4, 2.5, 3.6, 5.3, 7.2, 10.1, and 14.0 μm were injected with uniform spatial distribution and velocities calculated using the 1/7th power law at the inlet of the domain. 10,000 particles were injected for each diameter, resulting in a total of 70,000 particles per injection.

For the CFD models of nasal delivery, particles were injected into the flow domain at the inlet face with a blunt, random spatial distribution, according to the recommendations by Longest and Vinchurkar (2007). Each particle was assigned an initial velocity based on its spatial location, and the magnitude was determined according to the blunt velocity profile using the 1/7th power law, which generally approximates the velocity profile of an internal homogeneous turbulent flow (Hibbeler, 2018; White, 2006). The injection was generated with polydisperse particles to cover a range of diameters consistent with the experimentally determined particle size distribution (PSD). The represented particle size bins were 0.206, 0.533, 0.874, 1.498, 2.578, 4.218, 7.297, 11.632, and 13.850 μm. For each bin 3,000 particles were injected, which resulted in a total of 27,000 particles per injection. Particle number convergence was confirmed, as no noticeable change in the results was observed after doubling and tripling the number of particles per bin.

In order to report the particle deposition pattern, either a deposition efficiency (DE) or a deposition fraction (DF) was used. The DE of any particular region was defined as the ratio of the particle mass deposited to the particle mass entering that region expressed as a percentage:

(11) $$DE_{r}=\frac{Particle\;mass\;deposited\;in\;region\;r}{Particle\;mass\;that\;entered\;region\;r}\times 100\%$$

In the reported results, DE values in the PI and NT model are based on the mass of particles entering these regions, respectively. DE values in the filter are based on the mass of particles released from the device and also represent the lung transmission efficiency, also based on particle mass released from the device.

Deposition fraction for any region is defined as the ratio of the particle mass deposited in the region to the particle mass released from the device and expressed as a percentage:

(12) $$DF_{r}=\frac{Particle\;mass\;deposited\;in\;region\;r}{Particle\;mass\;released\;from\;device}\times 100\%$$

Based on these definitions, the sum of regional DFs equals 100%, while the sum of regional DEs does not. In this context, it is noteworthy that particles exiting the domain are assumed to deposit in the tracheal filter, and thus are included the sum of DFs to yield a total of 100%. DFs were only used in the final validation to directly compare the predicted results to those experimentally measured, in order to avoid scaling of the experimental values, which could lead to error propagation. In analyzing the impacts of the suggested model modifications on the deposition predictions, DE values were preferred because they are greater than or equal to DF values, which amplifies the differences in predicted particle deposition patterns among the compared cases.

### 2.4 Experimental methods

A mass of 10 mg of spray dried albuterol sulfate (AS) excipient enhanced growth (EEG) formulation was loaded in the air-jet DPI device (further details regarding the powder formulation are provided in the supplemental materials). Each device was actuated using a custom automated air source that allowed for a set flow rate to be delivered over a defined time period. In this case a relatively square flow profile was used with a Q90 flow rate of 3.9 LPM, where Q90 represents the volumetric flowrates (LPM), for which 90% of the rapidly sampled flowrate data of the measured waveform during the 10 mL air delivery lie below.

Particle size distributions for the devices were determined using a Next Generation Impactor (NGI), similar to Farkas et al. (2018a). Drug masses on each device component and on the NGI stages were assayed following washing with known volumes of deionized water and samples were quantified using High Performance Liquid Chromatography (HPLC). Further details regarding the HPLC method utilized in this study can be found elsewhere (Farkas et al., 2018b).

The NT model was assembled, connected to the Michigan test lung, and the lung was activated to start the breathing cycle. Next, the D2I device was connected to an automated (timer) air source (Howe et al., 2021), and inserted into one of the nostrils. The other nostril was held closed and the air source was actuated with the start of each inhalation, delivering 10 mL per actuation. The delivery process was repeated five times to empty the DPI of powder. The experiments were conducted with ambient air within the temperature-controlled laboratory (approximately 22 °C and 33.5% RH) and the airway walls were not prewetted. After each experiment, the DPI and nasal model were disassembled and washed using deionized water. Drug deposition on each part of the nasal model was determined using HPLC, as described in the previous study of Farkas et al. (2018b).

### 2.5 Case definitions

Table 1 summarizes all of the CFD cases that were considered in the present study. The variable parameters (columns) describe the selection of the k−ω model and its additional options, the applied NW corrections, and the EIM modifications. The first set of cases (1st four rows) are simulated with the default models available in the commercial package Ansys Fluent excluding all custom modifications via UDF. This explores the limits of the commercial package and the effects of the Default k−ω model variants. The second set of cases (rows 5–6) demonstrate the effects of recommendations regarding NW corrections previously published by Bass et al. (Bass, Boc, et al., 2019; Bass & Longest, 2018), and Thomas and Longest (2022). The remaining cases aim to incrementally evaluate the changes proposed in this study with an emphasis on NW corrections and EIM modifications. Hence, the next set of cases (rows 8–9) aims to demonstrate the effect of only applying the EIM modifications, and thus excludes all NW corrections. The final set of cases (rows 10–11) analyze the combined impact of the recommendations associated with the NW corrections as well as the EIM modifications on CFD predictions.

## 3 Results and discussion

### 3.1 Simulation with default Ansys Fluent settings

**Fig. 2:**
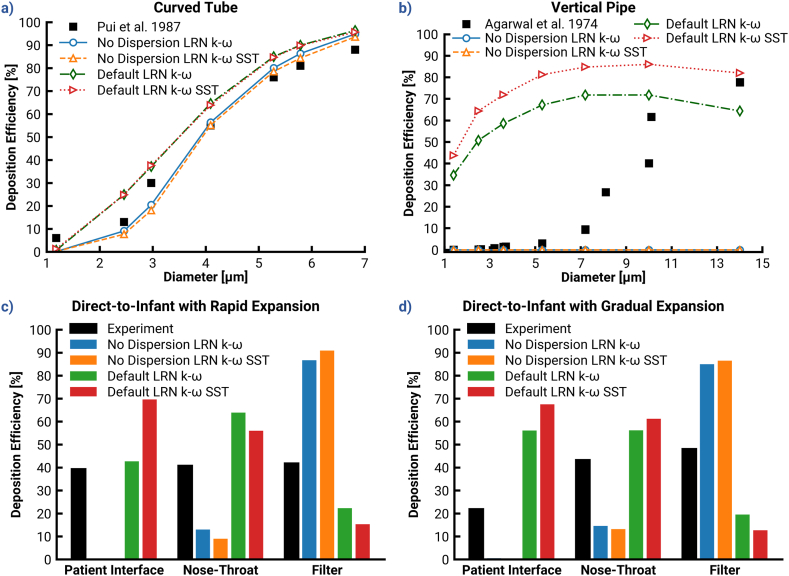
Demonstration of the two extreme bounds of deposition predictions possible in Ansys Fluent for all investigated cases with LRN k−ω and LRN k−ω SST turbulence models by comparison between the deposition fractions predicted with default Ansys Fluent models and the predictions without turbulent dispersion. Top: Deposition efficiencies in dependence of different classes of particle diameter for the (a) curved tube, and (b) the vertical pipe. Bottom: Local deposition efficiencies predicted for the direct to infant nasal delivery systems with different patient interfaces: (c) rapid expansion, and (d) gradual expansion.

In order to demonstrate the need for modifications, Fig. 2 provides a comparison between experimental deposition data and deposition simulated by the Ansys Fluent default settings, with and without the inclusion of turbulent dispersion of particle trajectories, for the LRN k−ω turbulence model variants (Cases 1–4). The difference in these simulated deposition profiles illustrates the range in which turbulent dispersion is responsible for deposition according to Ansys Fluent's present algorithm.

In Fig. 2a, it can be seen that deposition in the curved tube was governed mostly by impaction because with dispersion turned off the majority of deposition remained. For larger diameters, the limited effect of turbulent dispersion resulted in an accurate comparison with the experiment in the No Dispersion case. Conversely, regardless of turbulence model the Ansys Fluent Default case tended to over-predict dispersive deposition compared to the experiment, especially for smaller (1–4 μm) particles.

In the vertical pipe (Fig. 2b), the cases with dispersion turned off showed no deposition, as expected. With dispersion turned on, the Ansys Fluent Default case drastically over-predicted deposition of particles less than 10 μm. This discrepancy may be explained by the need for the default settings to cover a variety of engineering applications, many of which include particles larger than 10 μm. However, for simulations relevant to respiratory drug delivery, these benchmark cases make it clear that the Ansys Fluent Default tended to over-predict the deposition of relevantly-sized (1–4 μm) particles.

Regarding the effect of turbulence model selection, there was minimal influence on deposition in the curved tube, likely due to relatively low turbulence levels. Vertical pipe deposition was much more dependent on the selected LRN k−ω variant with a discrepancy of roughly 20% (absolute difference) between the alternatives.

Results in the D2I setups (Fig. 2c & d) (Cases 1–4) showed a similar trend, in that the Ansys Fluent Default cases (with turbulent dispersion) over-predicted deposition when compared with the values obtained by experiments. Overall, all default models provided poor agreement with the experimental data. It is noteworthy that both Ansys Fluent default variants predicted higher deposition in the GE than that in the RE, which is opposite to the trend seen in experiments. This indicates that the Ansys Fluent default models were unable to capture the distinctive influences of the gradual or rapid expansion on particle transport. The sum effects led to an underprediction of tracheal filter dose compared with the experimental target.

In contrast to the default cases, the simulations without any turbulent dispersion led to virtually no deposition in the PIs and very low deposition (below 15% compared to the experimental value of ≥ 40%) the in the NT region. This low deposition indicates that virtually all deposition in the PI is due to the turbulent dispersion, and that NT deposition is due to both impaction and turbulent dispersion.

The LRN k−ω SST model predicted higher turbulent deposition than the LRN k−ω variant, which had a lower rate of TKE production. In addition, the LRN k−ω model predicted a significantly longer inlet jet than the SST variant, particularly in the RE, which increased DE in the downstream NT region (additional evidence and associated discussion provided in the supplementary information). The LRN k−ω SST model was selected as the more desirable of the two variants because (a) its prediction of jet length was more aligned to experimental observations, and (b) the higher rate of deposition allows for greater control via NW corrections.

### 3.2 Simulation with existing recommendations

This subsection considers recommendations previously published by our group related to turbulence model selection and NW corrections. The specific model choices and parameters used in Bass Model (Bass, Boc, et al., 2019; Bass & Longest, 2018) and Thomas Model (Thomas & Longest, 2022) are provided in Table 1 (Cases 5–6).

**Fig. 3:**
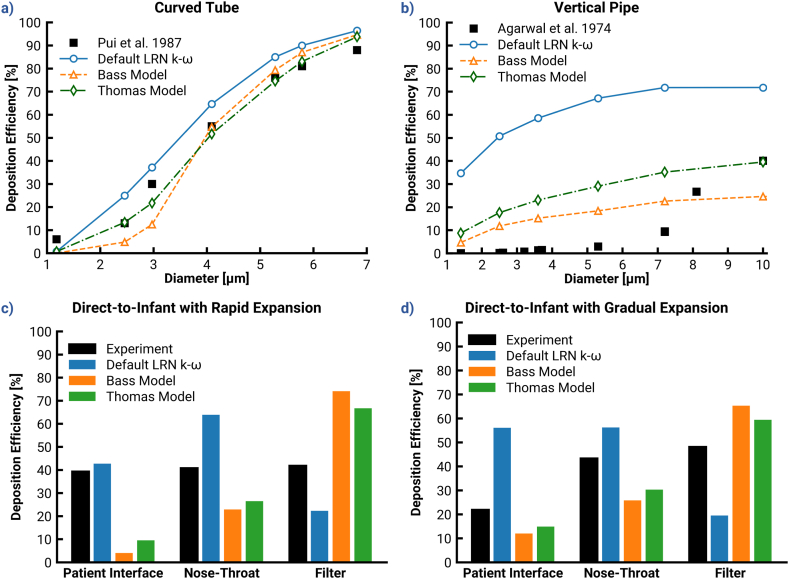
Demonstration of the effect of implementing the recommendations by Bass 2019 for all investigated cases. Top: Deposition efficiencies in dependence of different classes of particle diameter for the (a) curved tube, and (b) the vertical pipe. Bottom: Local deposition efficiencies predicted for the direct to infant (D2I) nasal delivery systems with different patient interfaces: (c) rapid expansion, and (d) gradual expansion.

Fig. 3 illustrates the depositional difference between the Ansys Fluent Default case and cases that use NW correction settings from the Bass and Thomas Models. The Thomas Model corrections appear to sufficiently match experimental results in the curved tube. The results from the Bass Model were originally intended for a hexahedral mesh, which explains the slight under-prediction when compared to the Thomas Model prediction. The curved tube case demonstrates the strengths of the corrections already in use, but these models did not consider the case of deposition by pure turbulent dispersion, as with the vertical pipe (Fig. 3b). Vertical pipe predictions from both the Bass Model and Thomas Model yielded a significant disparity from the experiment and failed to exhibit the depositional S-curve or closely match the DE at any particle size. However, they yielded a 25–50% increase in absolute accuracy compared to the Ansys Fluent Default, a result which emphasizes the need for NW corrections and also indicates the need for further improvements.

Considering the D2I setup, both the Bass Model and Thomas Model produced large discrepancies in deposition efficiency when compared to the target experimental data (see Fig. 3c & d). A $y^{+}\;limit$ of 60 with (Bass Model) or without (Thomas Model) the NW limit led to significant under-deposition in the PIs. This low deposition in the PI made more particles available for deposition in the NT region when compared to the default case. However, the applied NW corrections damped deposition in all model regions and yielded a filter dose that was markedly higher than that found in the experiments.

Although the Bass Model and Thomas Model addressed over-deposition in the PI and NT, they overdamped the NW turbulence and failed to match the experimental data. Hence, an improvement to the existing recommendations was necessary, with a focus on EIM modifications.

### 3.3 Modifications to the eddy interaction model

This section analyzes the contributions of Subsection 2.3.5. The first two changes to the EIM, presented as “EIM Modifications w/o DC” (Case 7), are the *variable interpolation* and *eddy lifetime lower limit*. The third modification, i.e., a *drift correction* (Mofakham & Ahmadi, 2020) is presented alongside the others as “EIM Modifications” (Case 8). It is noted that this section excludes NW corrections.

**Fig. 4:**
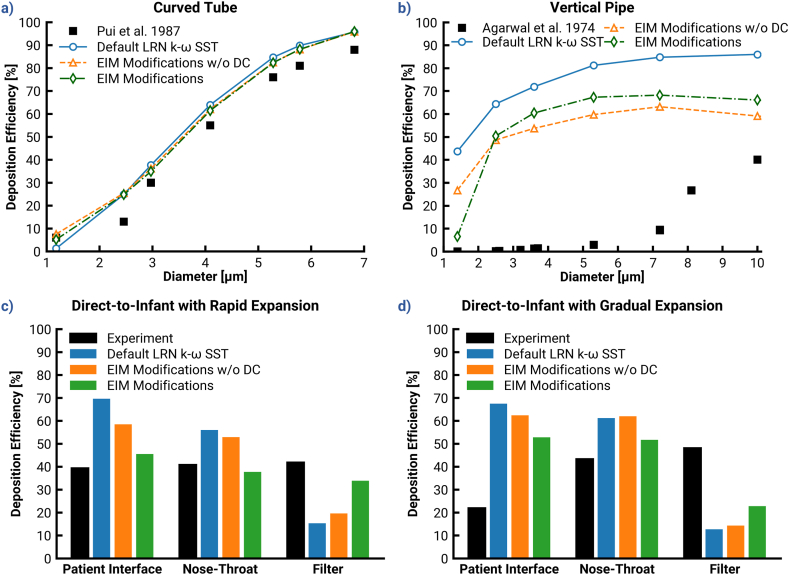
Demonstration of the effect of implementing the modified discrete random walk model on deposition predictions. Top: Deposition efficiencies in dependence of different classes of particle diameter for the (a) curved tube, and (b) the vertical pipe. Bottom: The local particle deposition efficiencies predicted with various models investigated in this study for the direct-to-infant (D2I) setups with different patient interfaces: (c) rapid expansion, and (d) gradual expansion.

Fig. 4 compares depositions predicted by the Ansys Fluent Default and EIM modifications, with and without DC (Cases 7 & 8) and with no NW corrections. In the curved tube, there was virtually no difference among the predictions with the EIM Modifications without and with DC, and the Ansys Fluent Default. The most notable difference between the Ansys Fluent Default and EIM Modifications was the 1.09 μm particle deposition. Neither the Bass Model nor the Thomas Model was able to match the 6% deposition of these particles, but the EIM modifications yielded higher accuracy at this particle size. In contrast, the vertical pipe case highlighted more distinct differences between the models. Generally, the EIM modifications improved agreement with the experimental data compared with the Default LRN k−ω SST model. Some of this benefit was counteracted by the DC, which increased deposition by 5–10% for all sizes except the 1.4 μm particles. Note that these cases evaluated modifications in the absence of the NW corrections, which induce the largest benefit towards agreement with experimental results.

The bottom panels of Fig. 4 (c & d) provide the predicted results for the D2I setups. The EIM modifications without DC noticeably improved deposition predictions (absolute differences of 11% in the RE and 5% in the GE), but still left a discrepancy of nearly 20% and 40% respectively. Although DE in the NT-regions also decreased, the reduction was less noticeable due to the influence of less deposition in the upstream component on that in the downstream region. Inclusion of the DC provided an additional reduction of approximately 10% and 13% DE in the RE and GE, respectively. NT deposition amounted to approximately 15% and 10% absolute DE with RE and GE, respectively. Note that the decrease may seem contrary to the increase observed in overall vertical pipe results, but the Mass Mean Aerodynamic Diameter (MMAD) of the D2I PSDs is below 2 μm so the two cases are consistent. The DC drove deposition for both interface cases closer to the experimental results when considering all regions together.

Despite the improvements achieved through updates to the EIM modifications, it is a challenging task to match the deposition in two different PIs. There is a need to slightly (<5%) decrease RE deposition and significantly (nearly 30%) reduce deposition in the GE to match the experiments. It is unlikely that identical NW corrections could yield these disparate reductions, so the distinctive features of the PI flow fields must determine NW correction settings (i.e., values of $y^{+}$ and NWlimits) required to attain experimental matches. Further analysis is required to identify a consistent means of inferring NW correction settings from case-specific flow fields.

### 3.4 Effect of variable near-wall corrections

To the authors’ knowledge, previous studies have applied $y^{+}\;limits$ as a constant throughout the domain. This may be a suitable method for cases consisting of consistent or semi-consistent flow regimes, but this study aims to improve the accuracy of models which span a range of turbulent, transitional, and laminar flows by applying variable $y^{+}\;limits$ within the domain. In order to demonstrate the range of turbulence in the D2I setups, the volume-averaged velocity magnitude and TKE from the RE, GE, and NT are shown in Table 2. As an illustration of how these quantities vary in the NW region, averages are presented for shells of the volume that stretch from the wall boundaries up to a given dimensionless height from the wall. The volume-averaged quantities were calculated by summing the products of the selected field variable (cell velocity, cell TKE) and cell volume in each cell under the given height and then dividing by the total volume of those cells. For example, the volume averaged TKE, $k_{avg}$ is calculated as follows:

(13) $$k_{avg}=\frac{1}{V_{y_{\lim}^{+}}}\;\int_{0}^{V_{y_{\lim}^{+}}}kdV=\frac{1}{V_{y_{\lim}^{+}}}\sum_{i=1}^{n}k_{i}\;V_{i}$$

where $V_{y_{\lim}^{+}}$ is the total volume of the cell zone defined by a range of dimensionless wall unit ($y^{+}$) values ranging from 0 to $y_{\lim}^{+}$, and $k_{i}$ and $V_{i}$ are the TKE and volume of individual cells. The results in Table 2 show that the TKE values differed by at least an order of magnitude between the PI and the NT region in the viscous ($y^{+}=5$) and buffer ($y^{+}=30$) layers as well as in the entire domain ($y^{+}=500$), while velocities in these regions differed by a factor of three-fold. The result is that the near-wall TKE and velocity magnitudes in these regions were very different, indicating significantly different levels of turbulence, which suggested that tuning parameters that damp the predicted turbulence should not be applied as a constant to all regions.

**Table 2:** Demonstration of the difference in the profiles of volume averaged velocity magnitude (Vel. mag.) turbulent kinetic energy (TKE) between different regions, i.e., patient interface (PI) and Nose-Throat (NT) in the calculation domain for the Direct-to-infant (D2I) delivery systems.

Case	Region	Flow Variable	Volume averaged through the dimensionless wall unit			
			$y^{+}=5$	$y^{+}=30$	$y^{+}=100$	$y^{+}=500$
D2I w/Rapid expansion	Patient interface	Vel. Mag. (m/s)	1.624	3.843	4.973	6.825
		TKE (m^2^/s^2^)	3.927	15.565	33.286	62.254
D2I w/Gradual expansion	Patient interface	Vel. Mag. (m/s)	1.897	7.790	11.481	11.974
		TKE (m^2^/s^2^)	2.694	11.639	17.849	19.383
All	Nose-Throat	Vel. Mag. (m/s)	0.215	1.264	1.961	2.067
		TKE (m^2^/s^2^)	0.023	0.276	0.489	0.527

**Fig. 5:**
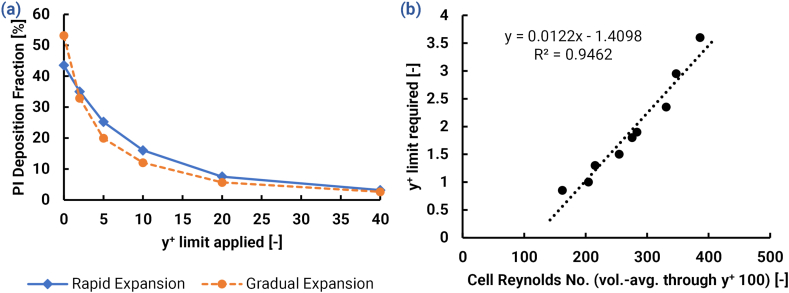
(a) The change in predicted deposition fractions in the patient interfaces (PIs) of the direct-to-infant setups in dependance of the various *y*^*+*^*limit* applied for the near-wall turbulence damping according to Matida et al. (2004) (b) The linear correlation between the *y*^*+*^*limit* required for the near-wall turbulence damping according to Matida et al. (2004) and cell Reynolds number volume averaged through *y*^*+*^ of 100. The points represent different y-plus limits required to match the experimentally measured deposition fractions in the expansion unit.

Variable $y^{+}\;limits$ in the PIs were examined in Fig. 5a and the predicted deposition fractions were heavily dependent on the selected $y^{+}\;limit$. The effect was similar in trend but different in both magnitude and slope for the two PIs until the curves converged at a $y^{+}\;limit$ of around 20, after which the effect of increase was nearly negligible. The asymptote occurred near 0% DE because these cases mostly contained turbulent dispersion rather than impaction, but other cases would likely asymptote at a higher DE that accounts for impaction.

To find a means of identifying case-specific and region-specific $y^{+}\;limits$, a CFD-predicted variable was sought that could serve as an independent variable in a quantitative correlation. Several flow field quantities, including the TKE, turbulent viscosity ratio (TVR), and velocity, were investigated and the most suitable candidate was the wall distance-based cell Reynolds number that was volume-averaged through a dimensionless wall-height of $y^{+}=100$. The following formula (Eq. 14) defines this special Reynolds number ${Re}_{i}$ for the cell i, where the distance from the closest wall $y_{i}$ was used as the characteristic length to avoid mesh dependence:

(14) $${Re}_{i}=\frac{y_{i}\;u_{f,i}\;\rho_{f,i}}{\mu_{f,i}}$$

(15) $${Re}_{cell,V_{avg}}=\frac{1}{V_{y_{\lim}^{+}=100}}\sum_{i=1}^{n}Re_{i}\;V_{i}$$

In these equations, $u_{f,i}$, $\rho_{f,i}$ and $\mu_{f,i}$ are the fluid velocity, density and dynamic viscosity in the cell centroid, respectively. Eq. (15) was used for the volume averaging of cell ${Re}_{i}$, where $V_{y_{\lim}^{+}=100}$ is the total volume of the cell zone between the wall and a dimensionless wall unit $y^{+}$ of 100. The upper boundary $y^{+}$ of 100 was chosen as the upper limit to definitively include the viscous and buffer layer not only for a developed flow but also for developing flow where the buffer layer can extend well beyond a $y^{+}$ of 30. Limiting the considered zone up to $y^{+}$ of 100 also ensured that core flow region was excluded, which is expected to have little influence on the wall deposition.

Fig. 5b presents the correlation found between the above defined ${Re}_{cell,V_{avg}}$ and the $y^{+}\;limit$ that was required for the CFD model to match the experimentally measured DF in multiple gradual and rapid expansions. For this purpose, multiple *in vitro* experiments were run with both of the PIs at various flow rates between 2 and 6 LPM for a polydisperse aerosol. This data covered a range of ${Re}_{cell,V_{avg}}$ from roughly 150 through 400, and an inlet Re range of roughly 3250 to 9750. The correlation fitted with the points by means of linear regression resulted in an $R^{2}$ value of 0.9462, indicating a good fit. Therefore, the following expression is recommended for estimation of $y^{+}\;limit$, which also sets any negative $y_{\lim,req}^{+}$ to zero:

(16) $$y_{\lim,req}^{+}=\max\left(0,0.0122\;{Re}_{cell,V_{avg}}-1.4098\right)$$

This correlation was applied to determine the $y^{+}\;limit$ for multiple systems and regions. For instance, the required $y^{+}\;limit$ in the RE and GE were determined to be 1.68 and 3.25, respectively. It is important to note that the volume over which the $Re_{cell}$ is averaged is dependent on both its height $\left(y^{+}=100\right)$, and its length, i.e., region covered. It is recommended that regions be divided based on changes in the flow field, which can be characterized by various flow field quantities such as velocity, TKE, TVR, and cell Re. For example, in the D2I setups, significant changes in the flow field were observed between the PI, nasal, and throat regions. Therefore, appropriate $y^{+}\;limits$ were set for each of these zones to accurately vary the extent of corrections applied to the NW turbulence.

Considering the nature of the mathematical function used for the correlation, it is noted that the linear regression used may not yield the optimum result. The exponential $\left(y_{\lim,req}^{+}=0.2912\;\exp\left[{Re}_{cell,V_{avg}}\right]\right)$ and quadratic $\left(y_{\lim,req}^{+}=3.7964\times 10^{-5}{Re}_{cell,V_{avg}}^{2}-8.7033\times 10^{-3}{Re}_{cell,V_{avg}}+1.2801\right)$ regressions offer better fits with $R^{2}$ values of 0.988 and 0.987, respectively. However, the linear approach was conservatively selected because (a) it would not yield exponentially large $y^{+}\;limits$ at ranges above the measured ${Re}_{cell,V_{avg}}$, if minor extrapolation is applied, and (b) only the linear correlation ensured $y^{+}\;limit=0$ for ranges well below the measured ${Re}_{cell,V_{avg}}$ (below 100), which is necessary for matching deposition in the NT region. This correlation can be extended or refined in the future as more experimental data becomes available over a broader range.

**Fig. 6:**
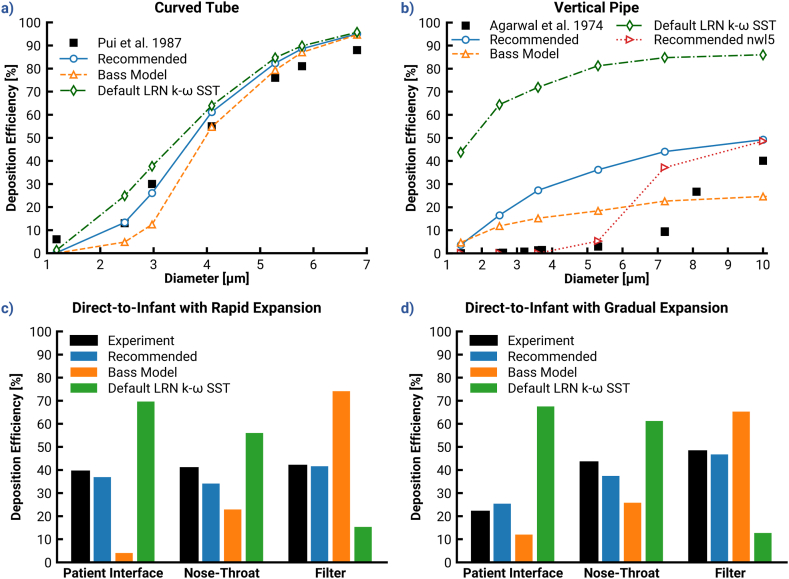
Demonstration of the effect of implementing all recommended settings and models devised in this study on the on the predicted deposition efficiency. Top: Deposition efficiencies in dependence of different classes of particle diameter for the (a) curved tube, and (b) the vertical pipe. Bottom: The local particle deposition efficiencies predicted with various models investigated in this study for the direct-to-infant (D2I) setups with different patient interfaces: (c) rapid expansion, and (d) gradual expansion.

The correlation between ${Re}_{cell,V_{avg}}$ and $y^{+}\;limit$ was applied and evaluated in both the benchmark geometries and the D2I setups; Fig. 6 shows the resultant deposition as “Recommended”. The correlation yielded a $y^{+}\;limit$ of 6 in the curved tube, and indeed a best-case match was produced. The Recommended model improved upon experimental agreement of the Bass and Thomas Models, and like them did not predict over-deposition of 2–3 μm particles as the Ansys Fluent Default did.

Within the vertical pipe, where deposition is highly sensitive to dispersion modeling, it is clear that neither the Recommended (without *NW-limit*) nor Bass Model was sufficient to provide a close match. However, the combination of the Recommended $y^{+}\;limit$ and a *NW-limit* of 5 μm was sufficient to achieve an improved match, especially for particle sizes <5μm. This is the only modification so far which managed to predict a depositional S-curve that was similar to the experiment, with little or no deposition for particles smaller than 5 μm.

The combined effects of LRN k−ω SST turbulence model, EIM Modifications, and the correlated $y^{+}\;limits$ (Case 9) greatly improved predictions of regional particle DEs in the D2I setups (shown in Fig. 6c & d), where the particle deposition is governed by a combination of turbulent dispersion and inertial impaction. A close match to the experimental results across the PIs (both D2I w/RE and D2I w/GE) and all local regions up to the filter transmission was achieved. This implies that the recommended method is effective in capturing the relevant physics and can be used to improve the accuracy of the predictions of aerosol deposition.

### 3.5 Final validation results: direct-to-infant aerosol delivery

**Fig. 7:**
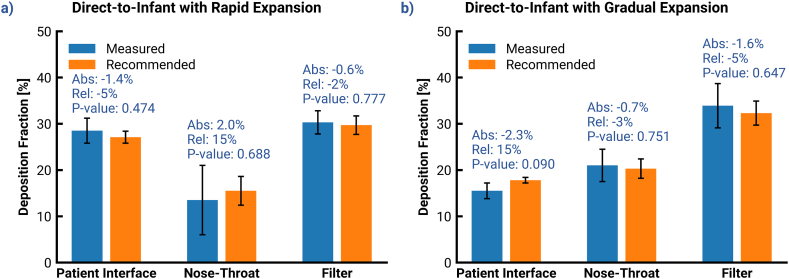
Comparison between the experimentally measured and CFD-predicted deposition fractions in various systems for delivery of DPI generated aerosol via nasal interfaces to a preterm infant nose-throat model; The four different systems are, (a) Direct-to-infant delivery via rapid expansion, (b) Direct-to-infant delivery via gradual expansion. (Abs = absolute difference between the means, rel: relative difference between the means, p-value: The probability of the differences being statistically insignificant obtained by means of a two-tailed *t*-test with 95% confidence interval).

**Fig. 8:**
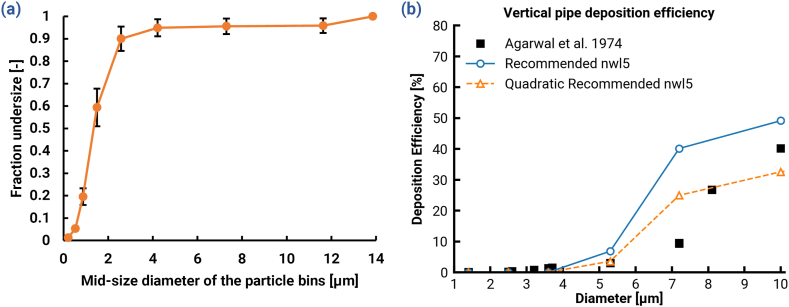
(a) Cumulative distribution of the initial particle sizes measured experimentally and used in the CFD simulations of the aerosol delivery to the infant nasal model. (b) A comparison of deposition in the vertical pipe using the $y^{+}\;\text{limits}$ predicted by the linear and quadratic correlations to the data presented in Fig. 6b.

In Table 3 and Fig. 7, a direct comparison is presented between the experimentally measured (*in* *vitro*) regional **deposition fractions** and those predicted by CFD for the two infant N2L systems: D2I with RE (see Fig. 7a) and D2I with GE (see Fig. 7b). The CFD-predicted deposition results were equivalent with the experimental data for both systems within the standard deviation of the measurements. The error bars on the CFD-predicted data are derived from the use of multiple PSDs. The measured MMAD at the inlet of the D2I systems varied slightly over three experimental runs (see Fig. 8a) with an average of 1.72μm and standard deviation of 0.14μm. Rather than averaging the PSDs, simulations were run using each PSD and these three runs were used to calculate mean and standard deviation (error bars) values. This approach allowed the standard deviation of the predicted CFD results to be calculated as well. Furthermore, a two tailed *t*-test with 95% confidence interval was performed to determine the existence of potential differences between the measured and predicted data, which may be statistically significant.

**Table 3:** Summary of the deposition fractions^a^ measured during all *in vitro* trials and those predicted by the CFD simulations with recommended models in this study for the direct-to-infant variants of the aerosol delivery to the preterm infant nasal model.

Location of deposition	Direct-to-Infant w/Rapid Expansion		Direct-to-Infant w/Gradual Expansion	
	*in* v*itro*	CFD	*in vitro*	CFD
**Capsule Retention (%)**	20.3 (11.1)	N/A	25.0 (5.2)	N/A
**Patient Interface (%)**	28.5 (2.7)	27.1 (1.3)	15.5 (1.7)	17.8 (0.6)
**Nose-Throat (%)**	13.5 (7.5)	15.5 (3.1)	21.0 (3.5)	20.3 (2.1)
**Filter (%)**	30.3 (2.5)	29.7 (2.0)	33.9 (4.8)	32.3 (2.6)
**Recovery (%)**	92.7 (0.8)	N/A	95.4 (0.5)	N/A

a Experimental mean values are presented for the mass percentages with respective standard deviations in parentheses.

The results showed that the absolute difference and absolute value of the relative difference fell within a range of 0.6%–2.3% and 2%–15%, respectively. These values were deemed acceptably low, as they satisfy the target accuracy of a maximum of 5% absolute error and 15% relative error between the predicted and experimental deposition fraction within each region, as set in the objective statement of this study. Moreover, the p-values for the two-tailed t-tests, which ranged from 0.09 to 0.78, were well above the limit of statistical significance (p = 0.05). Taken together, these findings provided strong evidence that the experimental and CFD predicted data sets were highly comparable. This demonstrates a satisfactory validation of the recommended set of CFD models employed in this study.

### 3.6 Limitations

Limitations of this study relate to approximating the *in* *vitro* setup, CFD model, and predictive correlation. Several assumptions were made in modeling the *in vitro* experiments. In the CFD model, an air-tight sealing was assumed between the PI and the NT model. This assumption is justified by the high recovery (92–95%) achieved in the *in vitro* experiments. Moreover, steady-state simulations at Q90 were used to represent an inherently transient process of air-jet DPI actuation. This assumption is reasonable because most of the aerosol mass is delivered around the peak of the actuation.

Further assumptions were associated with the representation of the flow field and particle trajectories. Primarily, while two-equation turbulence models are effective in modeling aerosol deposition in the upper airways, they are an approximation to a highly complex flow phenomena (Longest & Vinchurkar, 2007). Considering the discrete phase, a number of common cloud phenomena including inter-particle interactions (collision, nucleation, agglomeration, and break-up), which may significantly alter the PSD and deposition characteristics, were not considered. Hygroscopic growth was also ignored, but the very short particle residence time (∼0.05 s) and laboratory relative humidity (approximately 22 °C and 33.5% RH) support this approach. While we predict hygroscopic growth to occur quickly in the lungs (at RH ≈ 99.5%), our EEG formulations do not experience growth at typical ambient RH values (e.g. RH ≤ 50%). In keeping with common practice, this study neglected particle-wall interactions, re-aerosolization, and rolling of deposited particles. Additionally, two-way coupling of momentum between the discrete and continuous phase was not employed to account for any effect of the particles on the flow field. This is justified by an approximate estimate of the dilute discrete phase with an approximated low volume fraction based on aerosolizing 10 mg of powder with five boluses of 10 ml air volumes. Volume Fraction is observed to change over the air actuations and mixing occurs within the geometry as well. Currently, additional experimental data is not available to quantify the volume fractions to the level necessary that it can be included in the simulations. Furthermore, particle transport variables such as lift force, shape-effects, and Brownian motion were also neglected. This is justified by the emphasis on the handling of transport due to turbulent dispersion and based on a screening that revealed negligible contribution toward particle deposition results. Omission of the Brownian motion can be further justified because former work shows that deposition due to Brownian motion was insignificant for particles larger than 100 nm (Xi et al., 2008). Moreover, electrostatic forces, which could be an important factor, were not considered, as there was not enough data available on the complex charges on the aerosol particles. Despite these limitations, the assumptions made in this study resulted in an excellent agreement between CFD predictions and experimental data on a regional scale for the D2I aerosol delivery setups when including the developed NW corrections and EIM Modifications, which indicates that these assumptions were reasonable.

In the analysis of turbulence model selection, it was found that the LRN k−ω with SFC model was not ideal for simulations which include jet or high-shear conditions. This conclusion should be revisited in the future with *in* *vitro* data that measures jet length and shear behavior in additional domains. Such data would enable a more specific comparison between the predictions of turbulence models and may further support the decision reached by this study, which was made based on the single case of the RE PI.

Additionally, the presented correlation for $y^{+}\;limits$ is constrained by the limited data that was used to form it. Only two interfaces were analyzed and a limited range of ${Re}_{cell,V_{avg}}$ (150–400) was explored. Corrections for the vertical pipe were extrapolated up to ${Re}_{cell,V_{avg}}=600$ based on this dataset. The lack of data at higher values for ${Re}_{cell,V_{avg}}$ and the choice to use a linear fit are both limitations which represent a need for future work. Fig. 8 was included to show potential for the correlation to be improved. Fig. 8b illustrates that the fitting method (linear or quadratic) had a significant effect on the proposed result. The improved experimental match attained from the quadratic fit suggests that more work is required to encompass additional models with other delivery systems, interfaces, and flow rates.

## 4 Conclusions

This study investigated turbulence model selection and discrete phase model modifications to improve the accuracy of CFD predictions of particle deposition with RANS two-equation turbulence models in respiratory drug delivery systems. There were several key results which contributed to a successful validation of two D2I aerosol delivery models.

The first key result is that **the Ansys Fluent default model is inadequate for simulating turbulent dispersion of particles in the size range applicable for respiratory drug delivery** (less than 10 μm), **and at turbulence levels found within inhalers, interfaces and airways.** The model over-predicts turbulent deposition of particles in this size range, likely because of (a) the isotropic turbulence assumption required by two-equation turbulence models, and (b) the handling of small particles by the default EIM in regions of high specific dissipation.

The next contribution focused on turbulence model selection. The LRN k−ω model with SFC enhances the dissipation of k and dampens the dissipation of ω, resulting in a longer jet and lower dispersive deposition compared to the LRN k−ω SST model. The longer jet consequently increased DE in the NT-region of the D2I setups. By contrast, LRN k−ω SST model predicted high dissipation and shorter jet length consistent with experimental observations. This led to the second key result: **the LRN** k−ω **SST model outperforms the LRN** k−ω **turbulence model in capturing the turbulence features in a domain consisting of evolving flow regimes, especially those involving jets or other high-shear flows**. Unfortunately, application of this turbulence model alone did not increase the accuracy of deposition when compared to experiments. This discrepancy required further investigation of the EIM and led to new contributions.

The third key result is that **amendments to the default discrete random walk model, i.e., EIM Modifications, are necessary to improve accuracy of deposition predictions**. The *variable interpolation, eddy lifetime lower limit, and drift* *correction* improved filter deposition agreement with experiments by around 10% or more in both D2I systems and slightly increased the accuracy of deposition in the vertical pipe.

The last area of contribution is the new improvements to the NW corrections. A novel predictive correlation was developed that relates NW velocity, expressed as a volume-averaged cell Reynolds number in the boundary layer, to the $y^{+}\;limit$ required to match experimental deposition. This correlation can be used to quantitatively vary the extent of the NW turbulence correction throughout the computational domain. When the correlated tuning was applied in combination with the aforementioned improvements, deposition in the D2I setups was in excellent agreement with *in* *vitro* results, having absolute differences between 0.6% and 2.3%. This minimal error suggests that the model is capable of accurately predicting aerosol dispersion and transport in complex domains comprised of a variety of flow regimes. Therefore, the final key result for this study is that **tuning the** $y^{+}\;limit$ **to boundary layer velocity in different regions of a single simulation is crucial to achieving accurate deposition predictions in complex models**.

For the first time, a validated comprehensive model has been attained that begins with the highly turbulent air-jet outlet, includes a PI where transition to low level turbulence occurs, and also includes the NT region, where the flow regime continues to evolve. The presented methodology and the insights gained in this study will contribute to high-accuracy modeling of aerosol dispersion and transport through variable flow regimes, and support the design as well as optimization of pharmaceutical devices in respiratory drug delivery systems. Future model development work will focus on the accuracy of the LRN variants of the turbulence model against *in vitro* data from high-shear flows as well as application of the predictive correlation for NW corrections in other domains across a larger range of Reynolds Number to enhance robustness. Future work concerning the modeling approach will also compare the performance of the proposed methods to other established approaches such as the continuous random walk model.

## Declaration of competing interest

Virginia Commonwealth University is currently pursuing patent protection of DPI devices, patient interfaces and dry powder formulations described in this study, which if licensed and commercialized, may provide a future financial interest to the authors. WL is a member of the Journal of Aerosol Science Editorial Board.

## Data Availability

https://osf.io/jrmvf/?view_only=32736cef4c364785b645e406c5c56b64.

## Nomenclature

**Latin letters**
$a_{i}$: coefficients/constants (−)
$C_{c}$: Cunnningham correction factor (−)
$C_{D}$: drag coefficient (−)
$c_{l}$: Lagrangian time scale constant (−)
d: diameter (−)
f: drag factor (−)
G: randomly generated number with Gaussian distribution (−)
g: acceleration due to gravity (m s^−2^)
k: turbulent kinetic energy (m^2^ s^−2^)
L: Length (m)
m: mass (kg)
n: number of droplets (−)
Re: Reynolds number (−)
Stk: Stokes number (−)
$T_{l}^{+}$: Dimensionless Lagrangian integral time scale (s)
$T_{l}$: Lagrangian integral time scale (s)
t: time (s)
$u_{*}$: friction velocity (m s^−1^)
u: velocity (m s^−1^)
$\bar{u}$: mean velocity component in x direction (m s^−1^)
$\bar{u_{i}^{\prime n+1}}$: mean drift velocity (m s^−1^)
$u^{\prime }$: fluctuating velocity component in x direction (m s^−1^)
V: volume (m^3^)
$v^{\prime }$: fluctuating velocity component in y direction (m s-1)
$w^{\prime }$: fluctuating velocity component in z direction (m s^−1^)
y: normal distance from the nearest wall (m)
$y^{+}$: dimensionless wall unit (−)

**Greek letters**
Δ: difference (−)
ε: turbulence dissipation rate (m^−2^ s^−3^)
μ: dynamic viscosity (kg m^−1^ s^−1^)
ρ: density (kg m^−3^)
$\sigma_{i}$: RMS velocity fluctuation component (m s^−1^)
τ: relaxation time (s)
ω: specific dissipation rate (s^−1^)

**Subscripts**
D: drag
avg: average
cross: eddy crossing
e: eddy
f: fluid
lim: limit
m: mass
max: maximum
n: wall normal
p: particle
r: region
req: required

**Abbreviation**
AS: Albuterol Sulfate
CAD: Computer-Aided Design
CFD: Computational Fluid Dynamics
CRW: Continuous Random Walk
CT: Computed Tomography
D2I: Direct-to-Infant
DC: Drift Correction
DE: Deposition Efficiency
DF: Deposition Fraction
DNS: Direct Numerical Simulation
DPI: Dry Powder Inhaler
DPM: Discrete Parcel Method/Discrete Phase Model
DRW: Discrete Random Walk
EEG: Excipient Enhanced Growth
EIM: Eddy Interaction Model
GE: Gradual Expansion
HPLC: High Performance Liquid Chromatography
LES: Large Eddy Simulation
LPM: Liters per Minute
LRN: Low Reynolds Number
MMAD: Mass Mean Aerodynamic Diameter
N2L: Nose-to-Lung
NGI: Next Generation Impactor
NT: Nose-Throat
NW: Near-Wall
PI: Patient Interface
PSD: Particle Size Distribution
RANS: Reynolds-Averaged Navier-Stokes
RE: Rapid Expansion
RH: Relative Humidity
SDR: Specific Dissipation Rate
SFC: Shear Flow Correction
SIMPLEC: Semi-Implicit Method for Pressure Linked Equations-Consistent
SST: Shear Stress Transport
TKE: Turbulent Kinetic Energy
TVR: Turbulence Viscosity Ratio
UDF: User Defined Function
