# Supplementary material for: Development of an effective two-equation turbulence modeling approach for simulating aerosol deposition across a range of turbulence levels
Source: J Aerosol Sci. 2024 Jan;175:106262. doi: 10.1016/j.jaerosci.2023.106262 (PMC10698304; doi:10.1016/j.jaerosci.2023.106262)
Supplement: Multimedia component 1 [file mmc1.docx]

Supplemental Information for the Manuscript:

Development of an effective two-equation turbulence modeling approach for simulating aerosol deposition across a range of turbulence levels

Hasan Jubaer^[[1]](#footnote-1)^, Morgan Thomas^1^, Dale Farkas^1^, Arun V Kolanjiyil^1^, Mohammad A. M. Momin^2^, Michael Hindle^2^, Worth Longest^1,^^[[2]](#footnote-2)^^[[3]](#footnote-3)^*

# Materials and Methods

## Materials for Powder Formulation

Albuterol Sulfate (AS) USP was purchased from Spectrum Chemicals (Gardena, CA) and Pearlitol^®^ PF-Mannitol was donated from Roquette Pharma (Lestrem, France). Poloxamer 188 (Leutrol F68) was donated from BASF Corporation (Florham Park, NJ). L-leucine and all other reagents were purchased from Sigma Chemical Co. (St. Louis, MO).

Spray dried albuterol sulfate (AS) excipient enhanced growth (EEG) formulation was produced using the optimized method described by Son et al. (2013) using a Büchi Nano Spray Dryer B-90 (Büchi Laboratory-Techniques, Flawil, Switzerland). The EEG powder formulation contained a 30:48:20:2% w/w ratio of AS, mannitol, L-leucine, and Poloxamer 188. This powder is used as a model drug dry powder formulation, as it is readily available and easily quantifiable. 10 mg of powder was loaded into Quali-V^®^-I capsules donated by Qualicaps (Whitsett, North Carolina).

## *In vitro* Nose-Throat (NT) Model

The Nose-Throat (NT) model (see **Fig. S 1**a) used in this study was developed by our group in (first reported in Howe et al., 2022b) from CT scans of a 28-week old preterm infant provided by Dr. Robert M. DiBlasi from Seattle Children’s Hospital (Youngquist et al., 2013). The face of the model was cast using Dragon Skin™ 20 silicone (Smooth-On, Lower Macungie, PA) in a 3D printed mold (see **Fig. S 1**a & b). This flexible face was then fixed to a 3D printed section of the anterior nose, built using a Stratasys Objet24 3D Printer (Stratasys Ltd., Eden Prairie, MN) in VeroWhitePlus. The middle passage/nasopharynx section was built using stereolithography (SLA) in Accura ClearVue by Quickparts (Seattle, Washington). A filter adapter was built in VeroWhitePlus and attached to the pharyngeal outlet of the nasal model to estimate lung delivery.


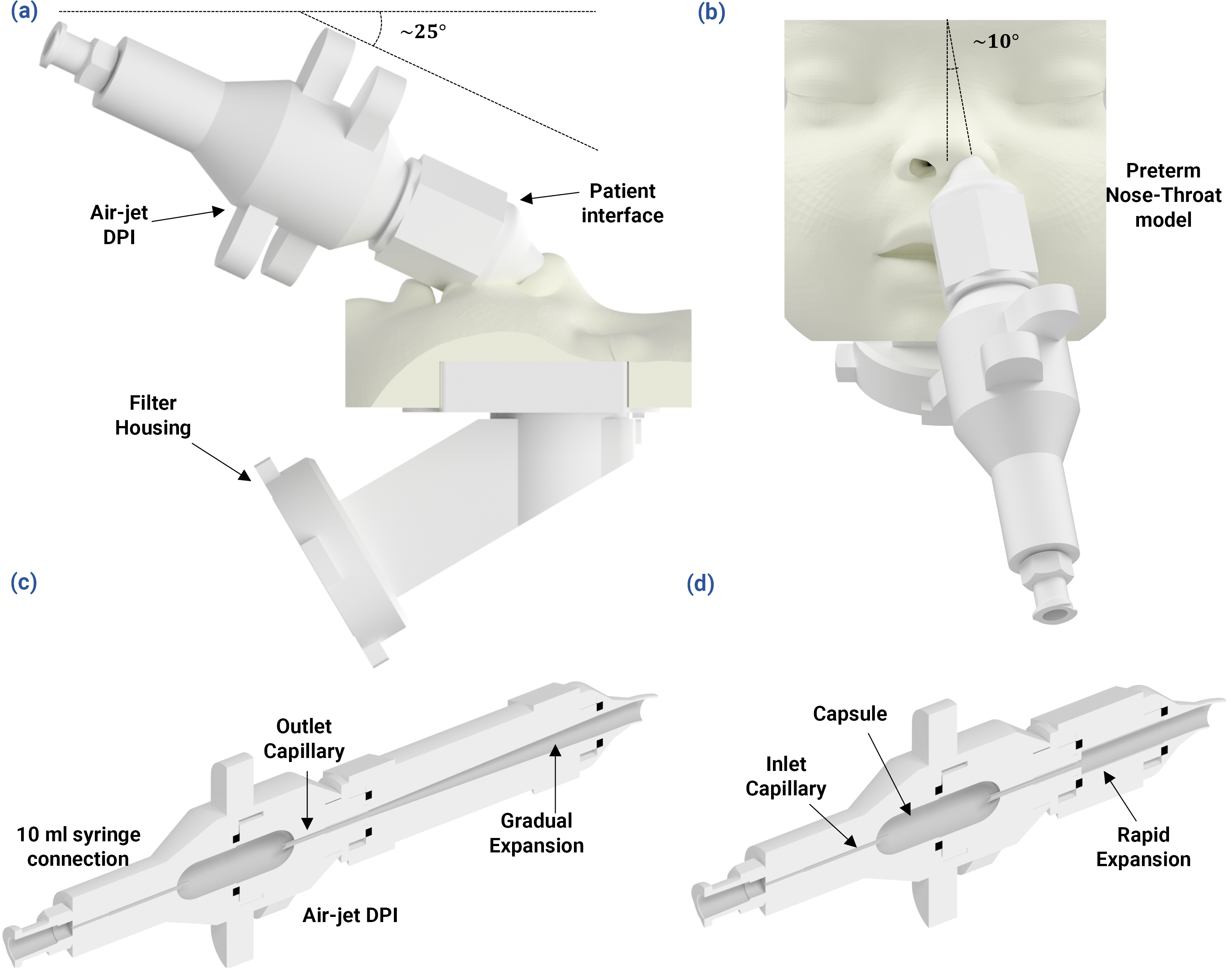


**Fig. S 1**: Overview of the direct-to-infant (D2I) aerosol delivery setup attached to a preterm infant nasal model. Panels (a) & (b) illustrates the side and top view of the setup, respectively. Panels (c) & (d) show cross-sectional isometric views of the 3D rendered patient interfaces used for the D2I aerosol delivery; (c) Air-jet DPI device attached to a gradual expansion (D2I w/ GE), (d) Air-jet DPI device attached to a rapid expansion (D2I w/ RE).

## *In vitro* Set-Up and CAD Assembly for CFD Model

**Fig. S 1a** shows the complete set up of the *in-vitro* trials. The air-jet DPI device used in this study was first developed by Farkas et al. (Farkas et al., 2018a, 2018b). A capsule chamber was designed to pierce a size 0 capsule with a sharpened inlet capillary with an ID of 0.6 mm and an outlet capillary of 0.89 mm. A gradual expansion (GE) or rapid expansion (RE) chamber was attached to the outlet of the capsule chamber. A curved nasal prong was connected to the outlet of GE or RE to direct the aerosol into the nose. The gradual expansion and curved nasal prong are similar to what was developed in Howe et al. (Howe et al., 2022a; Howe et al., 2022b).

For each *in vitro* PI-NT model assembly, images were taken of the experimental setup after the PI was inserted into the *in vitro* NT model. The angles at which the device was inserted, angle made by the device with respect to the plane perpendicular to the nasal palate and angle with respect to the plane of septum (as shown in **Fig. S 1**), and the depth of insertion, measured as the length of the device inside the nasal cavity beyond the nostril opening, were recorded using a combination of image analysis and visual inspection. These measurements were used to guide the device assembly with the NT model in the computational model. During the assembly, the CAD geometries of the *in vitro* hollow PI and NT models were first aligned by precisely matching the insertion angle and the insertion depth with the corresponding assembly measurements. This ensured that the computational model assembly closely replicated the *in vitro* model setup. Once the assembly of the PI and NT was complete, the air passage volume was extracted from both the CAD geometry of the hollow PI model and the NT model, and the assembly was consolidated into a single flow domain, assuming an airtight seal between the delivery prong and the nostril. Since the D2I delivery mode entails delivery through a single nasal cavity during the inhalation phase, only the pertinent nasal cavity was required for CFD model development and simulations. We compared the flow in a trimmed NT model (inactive nasal cavity was trimmed) assembled with the D2I-RE vs. a complete NT model (both nasal cavities untrimmed) assembled with the D2I-GE (as shown in Figure 1 in the manuscript), and found that the exclusion of the inactive nasal cavity had no adverse effect on the flow solution. The inactive nasal cavity was trimmed out during the computational model assembly with a wall boundary condition applied at the clipped surface for the other CFD model simulations. This approach helped to reduce computational time and resources, without compromising the flow solution.

## Meshing Strategies and Resolution

Meshes for all computational domains were generated by using the Meshing application embedded in FLUENT v2022R2 (ANSYS Inc., Canonsburg, PA, USA). Based on the advantages demonstrated by previous publications involving relevant geometries (Bass et al., 2019a; Thomas & Longest, 2022), the domains were discretized using unstructured polyhedral cell topology with prismatic cell layers in the near-wall region, following previously established best practices (Bass & Longest, 2018; Thomas & Longest, 2022). Appropriate near-wall resolution of the flow field was enabled by maintaining a wall $y^{+}$ value of approximately 1 across the domain (volume averaged wall $y^{+}$ was kept below 1 for all wall cells). The prismatic cell layer consisting of five cells in the NW region was constructed with a growth ratio of 1.2. The maximum skewness level for the surface mesh was kept below 0.4 and as an overall quality metric of the volume meshing a greater orthogonal quality than 0.25 was ensured.

To determine the optimum grid resolution, a mesh independence study was conducted on both D2I models i.e. D2I with rapid and gradual expansion. The study compared the volume average velocity magnitude, TKE, and deposition efficiency (DE) of different mesh resolutions. As for mesh resolutions, four successively increased resolutions ranging between 1.01 million and 7.89 million control volumes were considered for each model. These resolutions, i.e. coarse, medium, fine and extra fine meshes were obtained by roughly doubling the number of control volumes.

In **Fig. S 2**, the volume average velocity magnitude, TKE, and DE in the PI as well as in the filter are compared for a coarse (1.01 million cells), medium (2.07 million cells), fine (3.71 million cells), and extra fine (7.56 million cells) mesh of D2I with RE, and also a coarse (1.04 million cells), medium (2.15 million cells), fine (3.94 million cells), and extra fine (7.89 million cells) mesh of D2I with GE. The DE is calculated from particles that were exposed to the turbulent dispersion model, but no NW corrections or EIM modifications were applied.


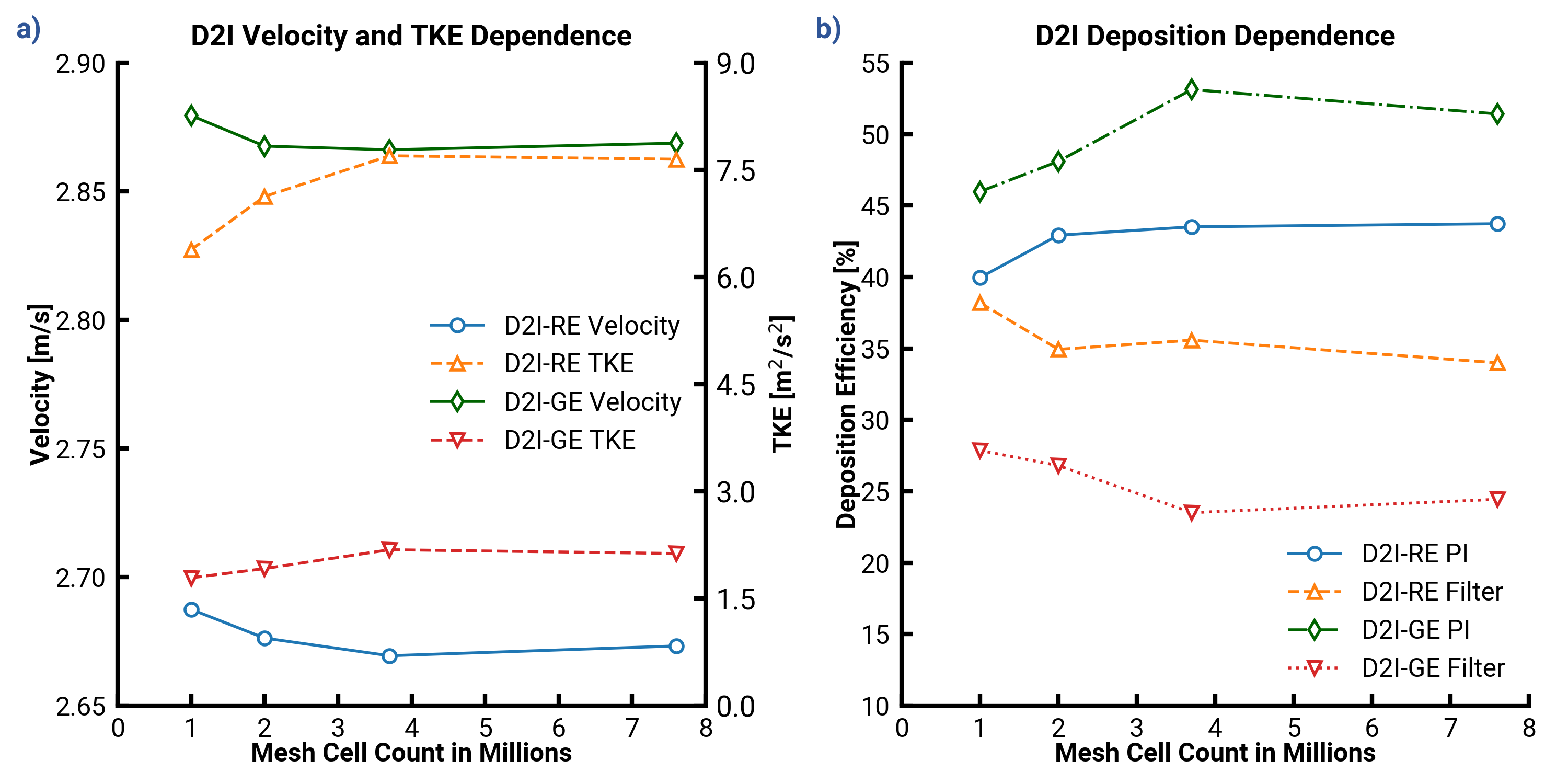


**Fig. S 2**: Summary of the mesh independence test conducted with both direct to infant (D2I) with rapid expansion (D2I-RE) and with gradual expansion (D2I-GE) models for key flow field variables and particle deposition behavior. Change in (a) volume averaged velocity magnitude and TKE, and (b) Deposition efficiency (DE) in the patient interface (PI) and in the filter for both D2I models with increasing mesh resolution.

The comparison revealed that the fine resolution of both D2I with RE (3.71 million cells) and D2I with GE (3.94 million cells) provide the best compromise between grid convergence and solution processing times. Between the fine and extra fine mesh resolution, the difference in predicted results is insignificant. Specifically, for the D2I with RE, the absolute value of the relative differences in volume average velocity magnitude, TKE, DE in the PI and in filter were 0.14%, 0.6%, 0.5%, and 4.7%, respectively. Similarly, these factors in the fine and extra fine mesh for the D2I with GE differ by 0.09%, 2.5%, 4.4% and 4% respectively. Therefore, the rest of this study uses the 3.71 million cell mesh for the D2I with RE model and 3.94 million cell mesh for the D2I with GE. These cell counts lie in the similar order of magnitude established elsewhere for studies of N2L aerosol delivery to a preterm NT model (Bass et al., 2022) and an infant nasal model via nasal interfaces (Bass et al., 2019b).

Thomas and Longest (2022) conducted a mesh dependence analysis for the curved tube (see published article for further details) and their converged mesh was used in this study. For the vertical pipe, mesh dependency was analyzed (see **Fig. S 3**) similar to the D2I models above. Four meshes with cell counts of 1.7, 3.6, 7.8, and 13.5 million cells were used to solve the flow field and values for volume-average velocity, TKE, wall shear stress, and particle deposition were compared. Concerning averaged velocity, the absolute values of relative differences for the first three meshes compared to the highest density mesh were 0.3%, 0.2%, and 0.1% (see **Fig. S 3a**). In a case as simple as the straight vertical pipe it makes sense that the volume-average velocity is stable for a large variation in mesh density. However, TKE variance was larger in magnitude at 18.9%, 7.8%, and 1.8% (see **Fig. S 3b**). Given that $y^{+}$ is a function of friction velocity, wall shear stress was also measured, which also showed a similar trend of convergence. In the highest density mesh the wall shear stress was measured to be 0.71 Pa and the relative differences of the other three meshes were 5.7%, 2.6%, and 0.9% (see **Fig. S 3c**). Concerning deposition, relative differences are not provided because they would have to be specific for each particle bin. However, it is clear that deposition in the 7.8 million cell mesh was quite close to that in the 13.5 million cell mesh (see **Fig. S 3d**). That result, combined with the relative errors of less than 2% for velocity, TKE, and wall shear stress were sufficient to conclude that the 7.8 million cell mesh was converged.


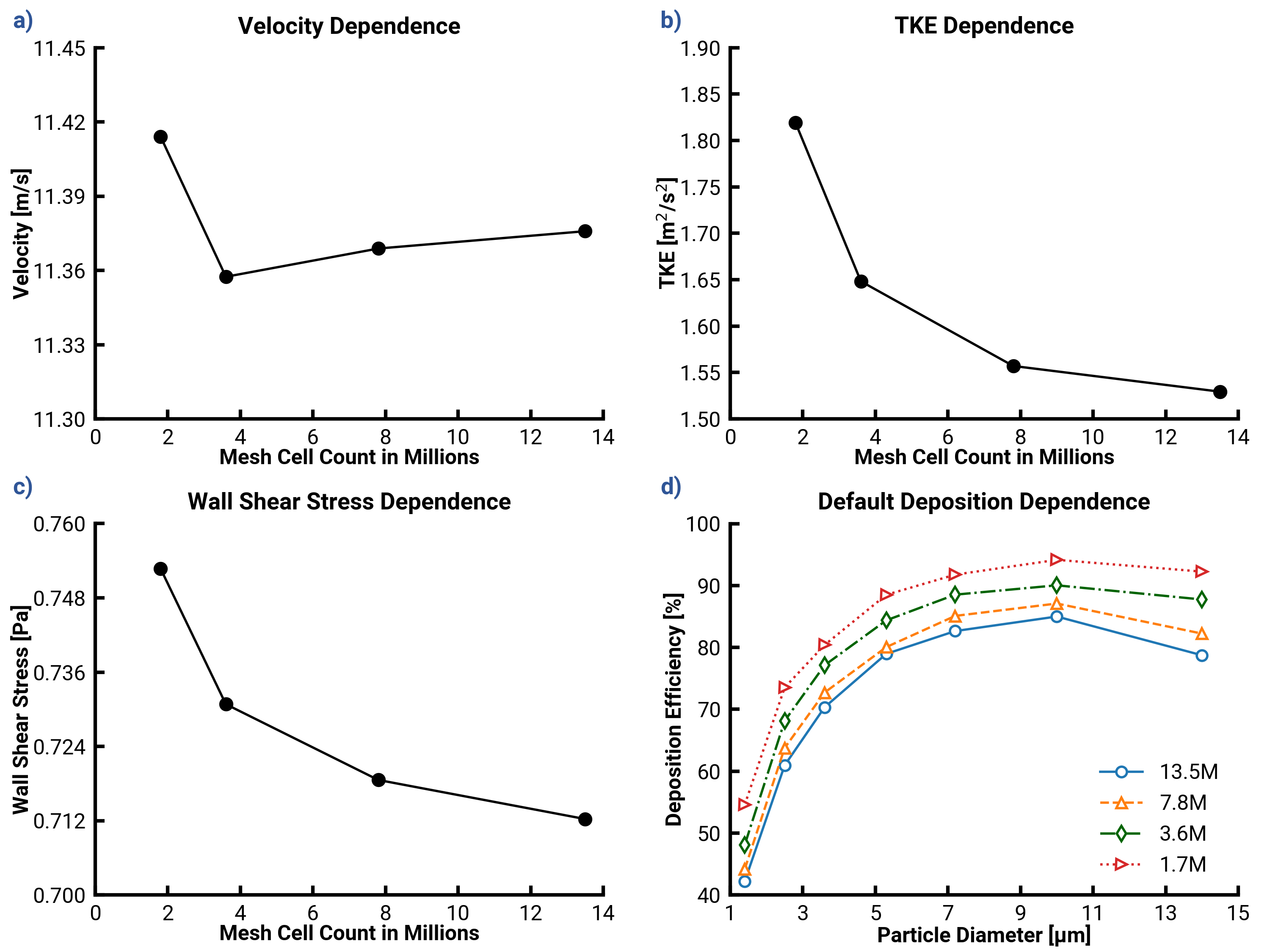


**Fig. S 3**: Summary of the mesh independence test conducted with the vertical pipe for key flow field variables and particle deposition behavior. Change in volume averaged (a) velocity magnitude, (b) TKE, and (c) wall shear stress with increasing mesh resolution for the vertical pipe; (d) Change in deposition efficiency (DE) of different particle diameter in the vertical pipe at various mesh resolutions.

# Additional Results and Discussion

## Analysis of Turbulence Model Selection

The turbulence model and its optional components play a crucial role in capturing flow field features. Therefore, we compared the LRN $k-\omega$ model with and without the SFC activated with the LRN $k-\omega$ SST model.

It is expected that differences between turbulence models will be most apparent in a developing flow rather than in a fully developed flow. It is a significant challenge to capture the behavior and dissipation of a jet with its surrounding free shear flow using two-equation models. Thus, the inlet jets in the PIs were chosen for a closer investigation. **Fig. S 4** and **Fig. S 5** demonstrate the differences of predicted flow development within the two PIs as the inlet jet dissipated along the axial coordinate up to a length of 40 inlet diameters, which necessitated extending the RE beyond its original length of 14.5 mm for generating data for this comparison only.


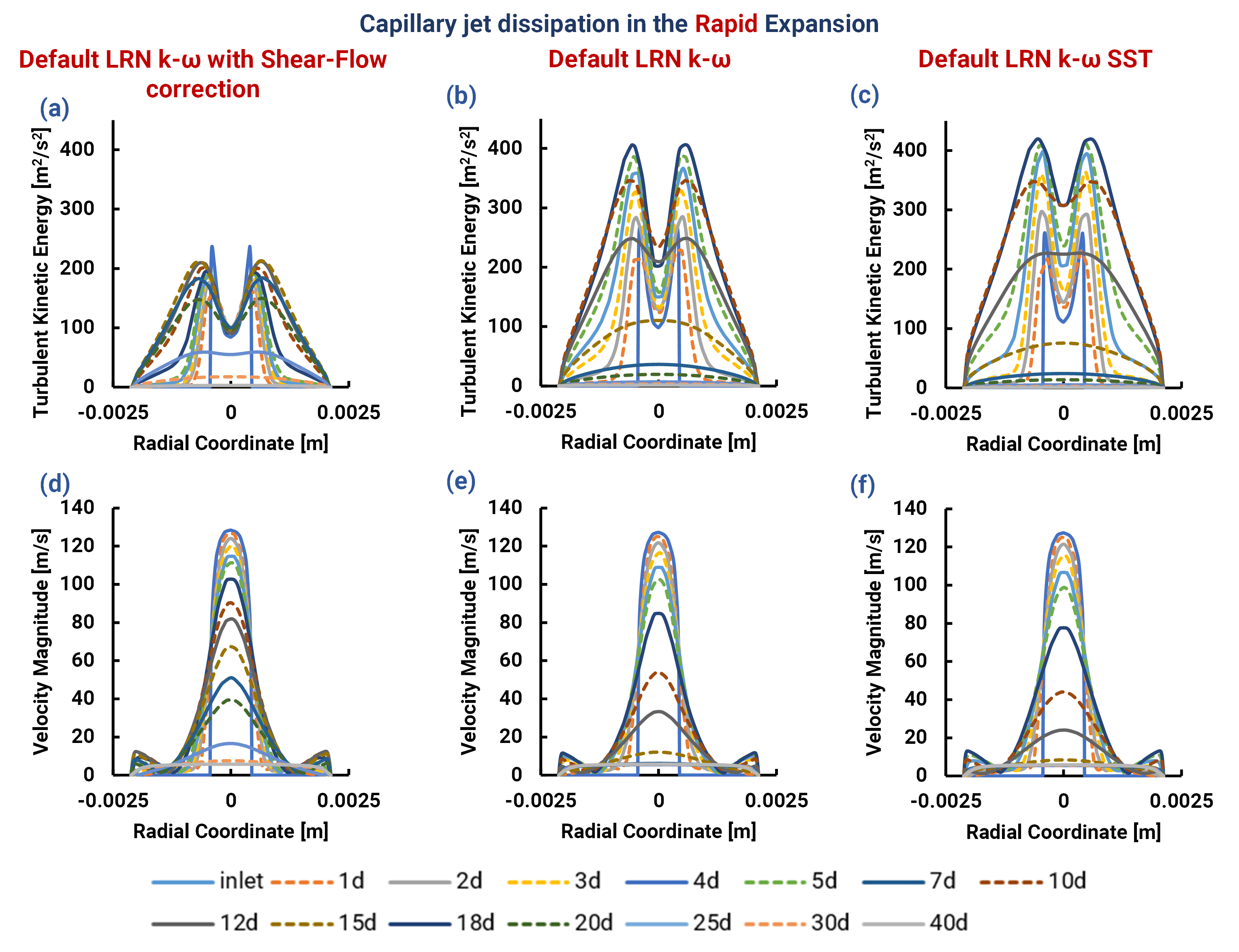


**Fig. S 4**: Development of a capillary jet entering a rapid expansion captured by the Default LRN k-ω (left panel with shear flow correction, SFC), the Default LRN k-ω (middle panel) without SFC, and the Default LRN k-ω SST (right panel). For comparison CFD model predicted axial profiles of the turbulent kinetic energy (a-c) and velocity magnitude (d-f) are plotted at various distance (multiples of the diameter d) from the inlet of the patient interface.

The key difference among the three turbulence models was the predicted length of the jet. The LRN $k-\omega$ model with SFC predicted a significantly longer length required for the dissipation of the jet than the LRN $k-\omega$ SST model. For instance, in the RE case (see **Fig. S 4**), it predicted a jet that extended almost up to 30d length. In contrast, the LRN $k-\omega$ SST model predicted no significant differences in the core velocity between the lengths of 15d and 40d away from the inlet, indicating that the jet is mostly dissipated at 15d length. In case of the GE (**Fig. S 5**), a fully developed flow could not occur in the expanding cross-section. However, it is still easily discernable that the LRN $k-\omega$ SST model attained a blunt parabolic velocity profile consistent with a turbulent flow without the presence of any jet at a distance between 12-15d, whereas the LRN $k-\omega$ model with SFC required a length of around 25d to achieve the same extent of jet dissipation. The profiles predicted by the LRN $k-\omega$ model without SFC approached those predicted by the LRN $k-\omega$ SST model, but predicted a relatively longer jet nonetheless. While direct flow field measurements were not available to compare the predicted results with experiments and thus screen the best performing model, the predictions made by the LRN $k-\omega$SST model could be corroborated by indirect evidence of particle deposition. Specifically, the RE was initially designed with a final length of 14.5 mm based on the minimum length required to allow the inlet jet to be fully dissipated, thereby preventing any additional depositional loss in the downstream components due to the inertial impaction of the jet. This experimental observation supported a quicker dissipation and shorter length of the jet, which was consistent with the predictions by the LRN $k-\omega$SST model.

The second significant difference is evident in the predicted profiles of TKE. The LRN $k-\omega$ with SFC predicted an enhanced dissipation of $k$ and a damped dissipation of $\omega$, causing the TKE profiles to be markedly lower in magnitude and gradient than the other two models. This difference was present for both PIs, although the RE showed a more significant difference than the GE. The key difference between the predictions by the LRN $k-\omega$ without SFC and the LRN $k-\omega$SST model was in the core region of the jet. This can be attributed to the fact that the LRN $k-\omega$ SST model utilizes the $k-\varepsilon$ formulation for the turbulent core and LRN $k-\omega$ formulation for the NW region. The difference in the gradients stemmed from the different cores predicted and the blending function used by the LRN $k-\omega$SST model.


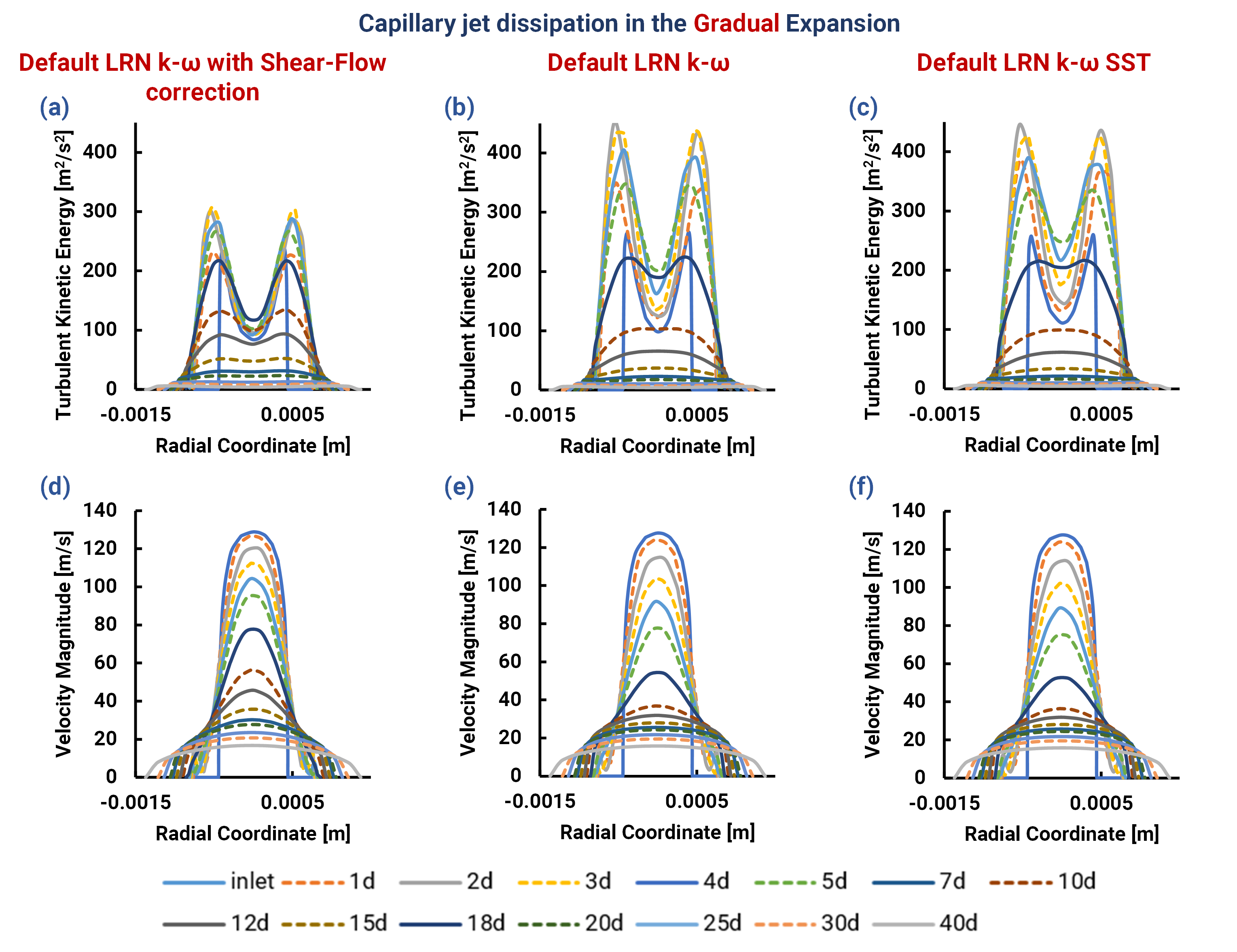


**Fig. S 5**: Development of a capillary jet entering a gradual expansion captured by the Default LRN k-ω (left panel with shear flow correction, SFC), the Default LRN k-ω (middle panel) without SFC, and the Default LRN k-ω SST (right panel). For comparison, CFD model predicted axial profiles of the turbulent kinetic energy (a-c) and velocity magnitude (d-f) are plotted at various distances (multiples of the inlet diameter d) from the inlet of the patient interface.

The differences in flow field predictions by various turbulence models are expected to have a direct impact on particle transport and deposition. The benchmark cases did not contain jets, so deposition comparisons in these cases (see **Fig. S 6a** & **b**) could be used to understand the effect of turbulence model selection in flows without that feature. In the curved tube (see **Fig. S 6a**), there was very little effect due to the shift to SST. It seems that particles larger than 4 µm experienced mild depositional shifts but they were not significant. Therefore, it may be asserted that impaction-driven deposition is not greatly affected by turbulence model selection. Conversely, there was a difference in the vertical pipe between the LRN $k-\omega$ and LRN $k-\omega$ SST cases, which disappeared if the SFC were toggled off (see **Fig. S 6b**).


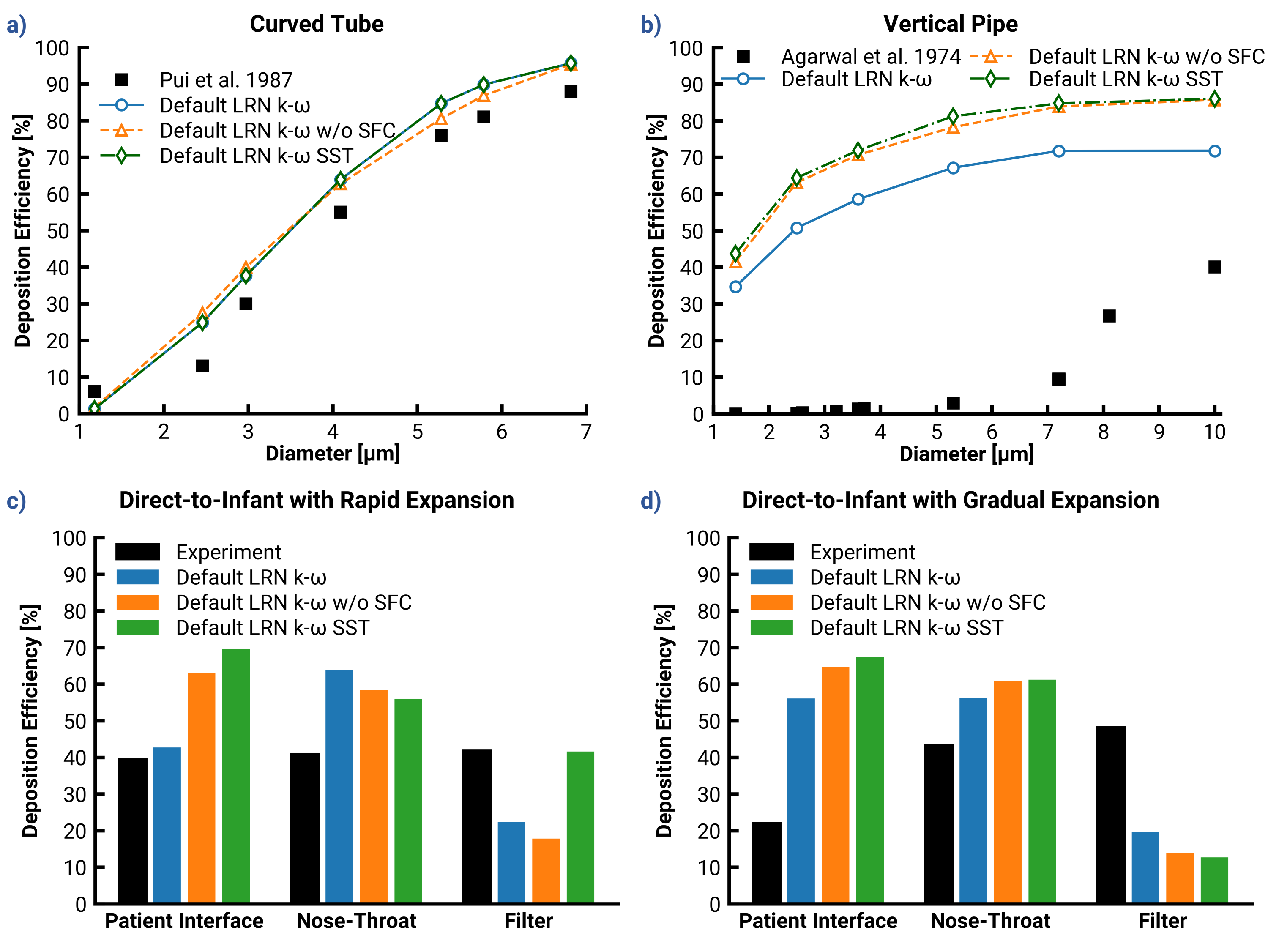


**Fig. S 6**: Demonstration of the effect of varying the turbulence model among LRN k-ω SST, LRN k-ω with and without shear flow correction (SFC) on the predicted deposition efficiency. Top: Deposition efficiencies in dependence of different classes of particle diameter for the (a) curved tube, and (b) the vertical pipe. Bottom: The local particle deposition efficiencies predicted with various models investigated in this study for the direct-to-infant (D2I) cases with different patient interfaces: (c) rapid expansion, and (d) gradual expansion.

Analyzing all particle deposition data presented in **Fig. S 6**, a general trend could be discerned. Selecting the $k-\omega$SST model led to the highest DE to be predicted, followed by the LRN $k-\omega$without SFC and the variant with SFC. Specifically, in the D2I cases with RE and GE, LRN $k-\omega$SST model resulted in approximately 26% and 13% higher DE compared to the LRN $k-\omega$variant with SFC, respectively. The single exception to this trend was observed in the NT region of the RE case, where a decrease in NT deposition efficiency of almost 8% was observed. This opposite trend can be attributed to the variation in the jet length predicted by the respective models. The LRN $k-\omega$model did not predict the jet to be fully dissipated after a length of only 14.5 mm within the RE, causing the residual jet to impact the back of the anterior nose. This caused an elevated DE in the NT-region, thereby overshooting the experimental value. By contrast, the prediction by the LRN $k-\omega$ SST model better matched the NT deposition. This finding was corroborated by the comparison of predicted deposition trends presented in **Figure 2** of the main manuscript, when turbulent dispersion was turned off, and only impaction-induced deposition was considered. It can be seen that the LRN $k-\omega$ variant predicted higher deposition in the D2I with RE case than the LRN $k-\omega$ SST model, which can be attributed to the sustained jet impacting the back of the nose.

## Effect of Drift Correction Term on Particle Concentration

The drift correction term proposed by Mofakham and Ahmadi (2020) is intended to counter the tendency of the conventional DRW to over-represent the concentration of low $Stk$ particles in regions of high $\omega$. They made note of the fact that particles dominated by turbulent dispersion are expected to have even concentration through at cross section in homogeneous internal flows such as the vertical pipe. To illustrate the effect of the drift correction term, **Fig. S 7** compares predictions of cross-sectional particle distribution in a slice of the vertical pipe. Panels (a) and (b) split the cross section into shells of equal area across the radius of the cross section and divide the number of particles present in each shell by the total number of particles present. For 1.4 µm particles (panel a), it is clear that the FLUENT Default predicted a dispersion where particles were largely concentrated at the center of the cross section and at its perimeter – more specifically under the $y^{+}=10$ boundary which roughly corresponds to the mid-point of a turbulent buffer layer. Conversely, the Modified DRW (EIM Modifications including Drift Correction) predicted a semi-even concentration of particles throughout the cross section and a decrease approaching the viscous sublayer. This effect is visualized in panels (c) and (d), where 1.4 µm dispersion can clearly be seen as somewhat uniform in the Modified DRW case and highly concentrated near the walls in the FLUENT Default case. Panel (b) shows that 10.1 µm particles were dispersed somewhat similarly by the FLUENT Default and Modified DRW cases. This is useful for demonstrating that particles with higher Stokes Number are not affected in a similar way by the addition of a drift correction term, which is expected. However, it is clear that FLUENT’s default settings predicted a particle concentration spike in the lower half of the buffer layer and in the viscous sublayer even for relatively large particles. This effect, demonstrated by both 1.4 and 10.1 µm particle distributions, may indicate that FLUENT’s DPM tends to push particles to the NW region and trap them there, which may partially explain why deposition predicted by FLUENT Default cases tends to be higher than experimental values.


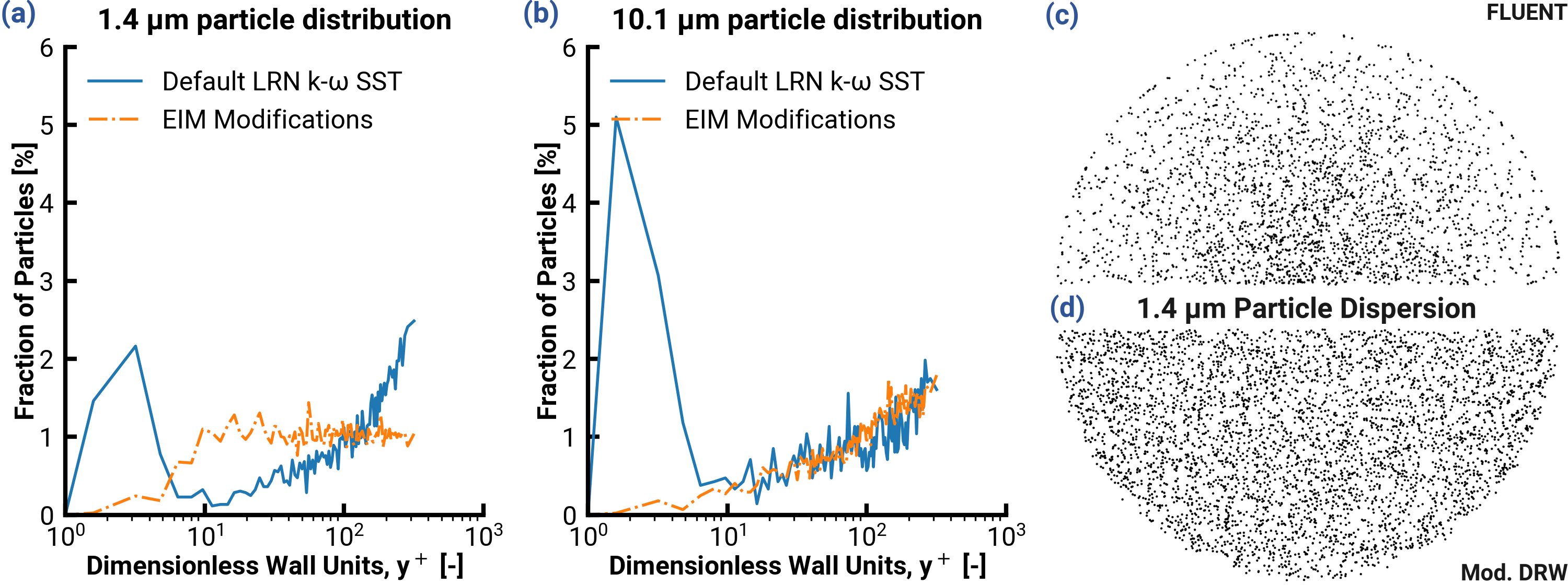


**Fig. S 7**: Demonstration of the effect of drift correction on particle dispersion. The left and middle panels show the area-based concentration profiles of (a) 1.4 µm, and (b) 10 µm particles plotted against distance from the wall. The right panel visualizes 1.4 µm particle dispersion in a slice of the vertical pipe predicted by the (c) FLUENT default, and (d) the modified discrete random walk model.

The overall decrease of particle concentration in the NW region consequently decreased the time required to simulate particle transport. This effect is an unintuitive result because cases with less deposition inherently require a larger average residence time. However, in this case, the reduction in simulation time by the decreased concentration of particles in the NW region overweighed the typical increase of simulation time due to less particle depositing that leads to higher average residence time. The authors believe this is an artifact of the selection of particle time step size, because FLUENT’s DPM mandates that the integration time step be limited by both cell resolution (i.e. distance to nearest cell boundary) and remaining eddy lifetime. The latter asymptotes to zero as a particle approaches a wall, so the decrease in simulation time is likely a combination of the decrease in near-wall concentration and the decision to limit the dimensionless Lagrangian timescale in the boundary layer to a minimum of 3 (see main manuscript section 2.3.5 for further information on EIM modifications).

# References

Bass, K., Boc, S., Hindle, M., Dodson, K., & Longest, W. (2019a). High-Efficiency Nose-to-Lung Aerosol Delivery in an Infant: Development of a Validated Computational Fluid Dynamics Method. *Journal of Aerosol Medicine and Pulmonary Drug Delivery, 32*(3), 132-148. <https://doi.org/10.1089/jamp.2018.1490>

Bass, K., & Longest, P. W. (2018). Recommendations for simulating microparticle deposition at conditions similar to the upper airways with two-equation turbulence models. *Journal of Aerosol Science, 119*, 31-50. <https://doi.org/10.1016/j.jaerosci.2018.02.007>

Bass, K., Momin, M. A. M., Howe, C., Aladwani, G., Strickler, S., Kolanjiyil, A. V., Hindle, M., DiBlasi, R. M., & Longest, W. (2022). Characterizing the Effects of Nasal Prong Interfaces on Aerosol Deposition in a Preterm Infant Nasal Model. *AAPS PharmSciTech, 23*(5), 114. <https://doi.org/10.1208/s12249-022-02259-z>

Bass, K. B., Susan, Hindle, M., Dodson, K., & Longest, W. (2019b). High-Efficiency Nose-to-Lung Aerosol Delivery in an Infant: Development of a Validated Computational Fluid Dynamics Method. *Journal of Aerosol Medicine and Pulmonary Drug Delivery, 32*(3), 132-148. <https://doi.org/10.1089/jamp.2018.1490>

Farkas, D., Hindle, M., & Longest, P. W. (2018a). Application of an inline dry powder inhaler to deliver high dose pharmaceutical aerosols during low flow nasal cannula therapy. *International Journal of Pharmaceutics, 546*(1), 1-9. <https://doi.org/10.1016/j.ijpharm.2018.05.011>

Farkas, D., Hindle, M., & Longest, P. W. (2018b). Efficient Nose-to-Lung Aerosol Delivery with an Inline DPI Requiring Low Actuation Air Volume. *Pharmaceutical Research, 35*(10), 194. <https://doi.org/10.1007/s11095-018-2473-7>

Howe, C., Momin, M. A. M., Aladwani, G., Hindle, M., & Longest, P. W. (2022a). Development of a High-Dose Infant Air-Jet Dry Powder Inhaler (DPI) with Passive Cyclic Loading of the Formulation. *Pharmaceutical Research, 39*(12), 3317-3330. <https://doi.org/10.1007/s11095-022-03409-5>

Howe, C., Momin, M. A. M., Bass, K., Aladwani, G., Bonasera, S., Hindle, M., & Longest, P. W. (2022b). In Vitro Analysis of Nasal Interface Options for High-Efficiency Aerosol Administration to Preterm Infants. *Journal of Aerosol Medicine and Pulmonary Drug Delivery, 35*(4), 196-211. <https://doi.org/10.1089/jamp.2021.0057>

Mofakham, A. A., & Ahmadi, G. (2020). Improved Discrete Random Walk Stochastic Model for Simulating Particle Dispersion and Deposition in Inhomogeneous Turbulent Flows. *Journal of Fluids Engineering, 142*(10). <https://doi.org/10.1115/1.4047538>

Son, Y.-J., Worth Longest, P., & Hindle, M. (2013). Aerosolization characteristics of dry powder inhaler formulations for the excipient enhanced growth (EEG) application: Effect of spray drying process conditions on aerosol performance. *International Journal of Pharmaceutics, 443*(1), 137-145. <https://doi.org/10.1016/j.ijpharm.2013.01.003>

Thomas, M. L., & Longest, P. W. (2022). Evaluation of the polyhedral mesh style for predicting aerosol deposition in representative models of the conducting airways. *Journal of Aerosol Science, 159*, 105851. <https://doi.org/10.1016/j.jaerosci.2021.105851>

Youngquist, T. M., Richardson, C. P., & DiBlasi, R. M. (2013). Effects of Condensate in the Exhalation Limb of Neonatal Circuits on Airway Pressure During Bubble CPAP. *Respiratory Care, 58*(11), 1840-1846. <https://doi.org/10.4187/respcare.02322>

1. Department of Mechanical and Nuclear Engineering, Virginia Commonwealth University, 401 West Main Street, P.O. Box 843015, Richmond, VA 23284-3015, USA [↑](#footnote-ref-1)
2. Department of Pharmaceutics, Virginia Commonwealth University, Richmond, Virginia, USA [↑](#footnote-ref-2)
3. * Corresponding author: [pwlongest@vcu.edu](mailto:pwlongest@vcu.edu) [↑](#footnote-ref-3)
